# Ontogenetic Shift in a Snake Venom Proteome Drives Distinct Proteomic Responses in Mouse Renal Mesangial Cells

**DOI:** 10.1021/acs.jproteome.6c00503

**Published:** 2026-10-15

**Authors:** Jorge da Cruz Moschem, Alison Felipe Alencar Chaves, Bianca Carla Silva Campitelli de Barros, André Zelanis, Solange M. T. Serrano

**Affiliations:** † Laboratory of Applied Toxinology, Center of Toxins, Immune-Response, and Cell Signaling (CeTICS), 534834 Butantan Institute, São Paulo, SP 05503-900, Brazil; ‡ Functional Proteomics Laboratory, Institute of Science and Technology, Federal University of São Paulo, UNIFESP, São José dos Campos, SP 12231-280, Brazil

**Keywords:** *Bothrops jararaca*, mass spectrometry, peptidomics, proteomics, SV40 MES13 cells, snake venom

## Abstract

*Bothrops* snakes are endemic to Latin America and are responsible for most medically relevant snakebites within the region. *Bothrops jararaca* venom proteome shows clear rearrangement upon neonate to adult transition. Similarly, the pharmacological activities displayed by the venom undergo a significant ontogenetic shift. This study evaluated the ontogenetic shifts in *B. jararaca* venom toxicity using SV40-MES13 cells to model murine glomerular nephrotoxicity. Cell viability assays revealed that adult venom possesses higher cytotoxic potency than newborn venom, reducing survival by over 30% at 4.0 μg/mL within 2 h, compared to just 15% for newborn venom. Label-free proteomics of cellular content and secretome across a concentration gradient demonstrated distinct, dose-dependent pathological mechanisms. At lower concentrations, adult venom induced a silent phase of molecular damage where cells remained viable but primed for apoptosis. Elevated concentrations of adult venom drove a pro-fibrotic phenotypic transition and extensive breakdown of extracellular matrix and cytoskeletal components, likely triggering detachment-induced anoikis. Conversely, newborn venom elicited a modest stress response. In the secretome, adult venom promoted extensive matrix degradation, whereas newborn venom increased adhesion proteins, and both venoms suggested activation of endogenous host proteases. This multicompartment approach provided a valuable framework for deciphering complex envenomation-induced tissue injury.

## Graphical abstract

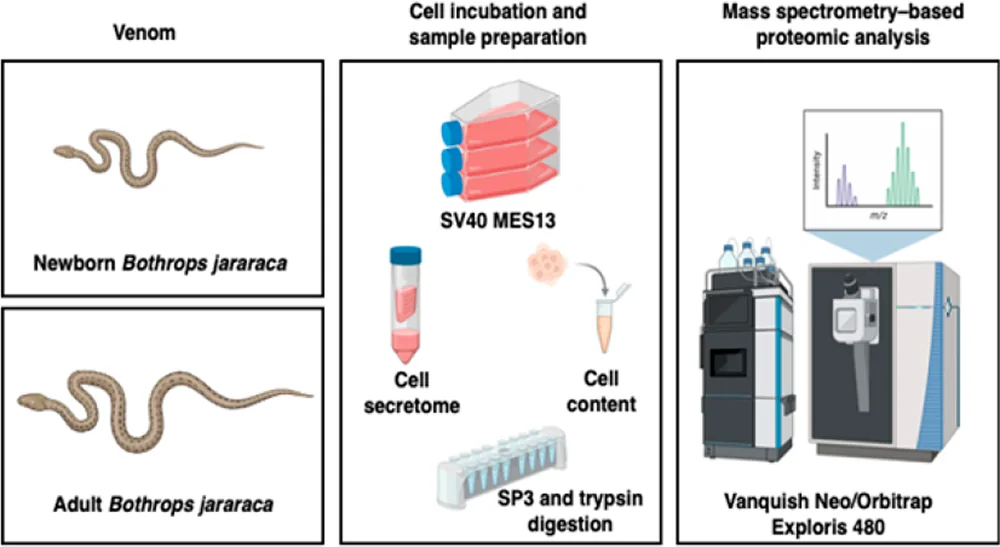

## 1 Introduction

Snake envenomation is classified by the World Health Organization as a neglected tropical disease, affecting thousands of victims annually worldwide. In Brazil, approximately 90% of snakebite accidents are caused by *Bothrops* snakes, due to their wide distribution across the territory and defensive behavior.^1^ *Bothrops* venoms are complex, composed of protein toxins and biologically active peptides.^2,3^ The main protein families identified in *Bothrops* venoms include (i) snake venom metalloproteases (SVMPs); (ii) snake venom serine proteases (SVSPs); (iii) phospholipases A_2_ (PLA_2_s); (iv) C-type lectins (CTLs); and (v) L-amino acid oxidases (LAAOs), among other less-abundant components.^2,4–6^

*Bothrops* envenomation is characterized by local and systemic effects. Local tissue alterations at the site of venom injection comprise pain, swelling, blisters, hemorrhage, and eventually necrosis.^1,7^ These are primarily caused by SVMPs, which are active upon extracellular matrix and adhesion proteins responsible for maintaining cell–cell and cell-basement membrane integrity.^8–10^ PLA_2_s also significantly contribute to tissue damage by hydrolyzing membrane phospholipids and releasing inflammatory mediators.^11,12^ Additional components such as hyaluronidases facilitate venom dissemination throughout tissues.^13^

Systemically, upon envenomation *Bothrops* venoms induce severe alterations in the bloodstream, including consumption coagulopathy, platelet dysfunction, endothelial damage, and the formation of microthrombi.^14,15^ These effects can lead to secondary complications in the lungs, brain, and kidneys.^16–19^ Among the systemic manifestations, acute kidney injury (AKI) is a frequent cause of mortality in patients who survive the initial phase of severe envenomation. Due to the kidneys’ high vascularization and filtration role, they are particularly vulnerable to injury. AKI occurs in up to 38% of *Bothrops* snakebite patients, depending on factors such as age, venom dose, and delays in receiving medical treatment, with a mortality rate ranging from 13% to 19%.^20,21^

In *Bothrops jararaca*, a snake widely distributed in the Central, Southeastern, and Southern regions of Brazil and largely responsible for snakebites in this highly populated area, the ontogenetic variation of the venom proteome is well characterized, with a clear rearrangement upon neonate to adult transition.^22–25^ In general, newborn snakes have venom with higher coagulant activity compared to adults. Likewise, the LD50 (median lethal dose) of newborn venom is lower in birds. In mammals, this trend is reversed as adult venom exhibits higher toxicity, characterized by a decrease in LD50 value compared to juvenile specimens.^23,26^ These studies have correlated this rearrangement in the framework of toxins composing the venom directly with the feeding habits in each stage of the snake life. To illustrate, the diet of juvenile specimens consists of small arthropods and lizards, while the adult phase is characterized by a preference for endothermic rodent mammals.^27,28^

The clinical presentation of *B. jararaca* envenomation varies significantly between newborn and adult specimens.^14,15,29^ Moreover, a comprehensive study reported that newborn venom possesses reduced proteolytic, hemorrhagic, edematogenic, ADPase and 5′-nucleotidase activities. Conversely, newborn venom is characterized by increased levels of L-amino acid oxidase and hyaluronidase activities, as well as higher potency in inducing platelet aggregation, procoagulant effects, and protein C activation.^22^

Recently, we reported an extensive study of the renal effects of adult *B. jararaca* venom in a murine model. By identifying primary altered pathways by proteomic, degradomic, and phosphoproteomic analyses, we shed light on both the direct and indirect mechanisms of toxin activity and identified potential therapeutic targets.^30^ Given that newborn *B. jararaca* venom significantly impacts hemostasis and the kidneys serve as primary filtration sites, this study compares the effects of newborn and adult venoms on the renal mesangial cells. Using mass-spectrometry-based proteomics, we aimed to characterize the metabolic and signaling pathway alterations triggered by these toxins.

## 2 Materials and Methods

### 2.1 *Bothrops jararaca* Venom

Newborn venom: lyophilized venom samples from 694 2-week-old newborns (359 male and 335 female specimens) born from snakes bred in captivity at the Herpetology Laboratory of Instituto Butantan.^23^

Adult venom: lyophilized adult venom was composed of samples from 888 specimens of unknown age and sex collected in Southeastern and Southern regions of Brazil, and it was provided by the Herpetology Laboratory at Butantan Institute (São Paulo, Brazil).

The venom samples were resuspended in 150 mM NaCl and kept in ice for resolubilizing, then centrifuged for 10 min at 12,000*g*, at 4 °C, and subjected to protein content determination using the BCA reagent (Thermo Scientific).

### 2.2 Animal Ethics Statement

The venoms used in this study were collected around 2008 at the Butantan Institute. At that time, the regulations governing the use of animal venoms for research purposes did not require approval by an ethics committee in the same manner as currently required in our institution. Therefore, the present study made use exclusively of venom samples that had already been collected and were available for research at the Butantan Institute, without any new venom collection being performed as part of this study.

### 2.3 Cell Culture

Experiments were performed using the SV40 MES 13 cell line (ATCC CRL-1927), an immortalized glomerular mesangial cell line derived from 7- to 10-week-old *Mus musculus*. Cells were confirmed mycoplasma-free and used at passage 32. Cells were maintained in DMEM (Dulbecco’s modified Eagle’s medium), supplemented with 10% fetal bovine serum (FBS), 1% penicillin and streptomycin (complete medium) in an incubator at 37 °C, in a humidified atmosphere, with 5% CO_2_.

### 2.4 Cell Viability Assay with Newborn and Adult *B. jararaca* Venom

SV40 MES 13 cells were seeded in 96-well plates at an approximate density of 10^4^ cells/well (100 μL) and maintained in complete DMEM for 16 h in an incubator at 37 °C, under 5% CO_2_. For the viability assay, complete DMEM was removed, cells were washed three times with PBS (phosphate buffered saline) and replaced with DMEM without FBS and phenol red (100 μL) and incubated for 16 h. Then cells were incubated with DMEM without FBS and phenol red (100 μL) containing five concentrations of newborn or adult *B. jararaca* venom (0.25, 0.5, 1.0, 2.0, and 4.0 μg/mL) or with 150 mM NaCl (control) for 2 h at 37 °C, under 5% CO_2_. The experiment was performed in six technical replicates for each condition. The culture medium was removed, and 100 μL of MTT (3-[4,5-dimethylthiazol-2-yl]-2,5 diphenyl tetrazolium bromide) solution (Sigma-Aldrich) at 0.5 mg/mL were added to each well. After 2 h, the MTT solution was removed and the formazan crystals formed were solubilized in 150 μL 100% DMSO, and cell viability was calculated by the absorbance of the solution, determined in a spectrophotometer at a wavelength of 570 nm, comparing the values of the viability of control cells (no venom) and those incubated with venom.

A *t* test with a family wise error correction using Holm-Bonferroni for multiple comparisons was performed to determine the statistical significance of the effects of the venoms on the cells.

### 2.5 Incubation of SV40 MES 13 Cells with Newborn and Adult *B. jararaca* Venom and Sample Collection for Proteomic Analysis

SV40 MES 13 cells were seeded in Corning’s enhanced T-75 Flasks at a density of 5 × 10^6^ cells/flask (10 mL/flask) and maintained in complete DMEM at 37 °C, under 5% CO_2_. Before the incubation with *B. jararaca* newborn and adult venom, complete DMEM was removed, the cells were washed three times with PBS and replaced with DMEM without FBS and phenol red (10 mL/flask) and the cells were incubated for 16 h. Then cells were incubated with DMEM without FBS and phenol red (10 mL/flask) containing five concentrations of newborn or adult *B. jararaca* venom (0.25; 0.5; 1.0; 2.0 and 4.0 μg/mL) or with 150 mM NaCl (control) for 2 h at 37 °C, under 5% CO_2_, in triplicate. Proteomic analysis of cellular content and conditioned medium (secretome) was also performed in triplicate, resulting in a total of 216 samples ().

### 2.6 Cell Content and Secretome Preparation for Proteomic Analysis

After cell incubation with 150 mM NaCl, or newborn or adult *B. jararaca* venom, the secretome was obtained by collecting the total cell conditioned medium and subjecting it to filtration using 0.22 μm syringe filters followed by separation using 3 kDa molecular mass cutoff filters (Amicon) under centrifugation at 3,000*g* and 4 °C. The retained fraction (>3 kDa concentration) was used in all subsequent experiments, and its protein concentration was quantified using the BCA reagent (Thermo Scientific).

The cells were subjected to lysis by adding 250 μL per flask of a buffer containing 100 mM HEPES (hydroxyethyl piperazineethanesulfonic acid), 1% SDS (sodium dodecyl sulfate), and 50 mM EDTA (ethylenediaminetetraacetic acid)) followed by vortexing steps interspersed with resting steps on ice. Subsequently, the mixture was centrifuged for 10 min at 12,000*g*, 4 °C, and the supernatant was recovered and subjected to protein quantification using the BCA reagent (Thermo scientific).

A total of 50 μg of proteins from the cellular content and secretome samples were submitted to single-pot, solid-phase-enhanced sample preparation (SP3) protocol.^31^ Briefly, protein disulfide bonds were reduced with 5 mM tris­(2-carboxyethyl)­phosphine (TCEP) and alkylated with 20 mM 2-chloroacetamide (CAA) for 30 min at room temperature, in the dark. A mixture of hydrophobic:hydrophilic magnetic beads (Sera-Mag SpeedBeads, Cytiva) at a 1:1 ratio was washed with water three times and resuspended in the original volume. Samples of reduced and alkylated proteins were incubated at a 1:10 ratio of proteins:beads and an equivalent volume of ethanol was added to reach a concentration of 50% ethanol in the solution. Samples were then incubated for 5 min under shaking at 1,000 rpm at room temperature. After this period, the tubes containing the proteins and beads were placed on a magnetic rack for 10 min. The supernatant was discarded, and the beads were washed five times with 80% ethanol, in cycles of resuspension and incubation on the magnetic rack. Protein digestion was performed using trypsin (Sigma-Aldrich) at a 1:100 enzyme to substrate ratio for 16 h at 37 °C and 1,200 rpm in a 50 mM ammonium bicarbonate solution. Afterward, the supernatant was collected and transferred to a LoBind protein tube, and the peptides were dried by vacuum centrifugation and stored at −80 °C until LC-MS/MS analysis.

### 2.7 LC-MS/MS

Tryptic peptides (300 ng) derived from cellular content and secretome were resuspended in 25 μL of 0.1% formic acid and sonicated for 15 min prior to LC-MS/MS analysis. Peptide separation and analysis were performed on a Vanquish Neo/Orbitrap Exploris 480 system (Thermo Scientific) equipped with a commercial capillary precolumn (PepMap Neo, 5 mm, 5 μm C18) and an analytical column (PepMap Neo, 150 mm, 2 μm C18). The Nanospray Flex was operated at 270 °C and 2.4 kV and coupled to a FAIMS Pro Duo interface (High Field Asymmetric Waveform Ion Mobility Spectrometry) with two compensation voltages (−45 V and −60 V). The mass spectrometer was operated in Data Independent Acquisition (DIA) mode with a 4 *m*/*z* isolation window covering the 430–650 *m*/*z* range for label-free quantification experiments.

The default precursor charge state was set to +2, and ions with unassigned or singly charged precursors were excluded. Stepped normalized collision energies of 32% and 36% were used, with a maximum injection time of 50 ms, and MS1 and MS2 scans were acquired at resolutions of 60,000 and 30,000, respectively. Peptide separation was carried out using a specific gradient for each type of sample: for (i) cellular content, a 120 min gradient (5–30% buffer B, composed of 80% acetonitrile and 0.1% formic acid in buffer A, composed of 0.1% formic acid, over 99 min, 30–40% over 9.5 min, 40–99% over 0.2 min, and held at 99% for 10.9 min at a flow rate of 300 nL/min); for the (ii) secretome, a 60 min gradient (5–30% buffer B over 49.7 min, 30–40% over 4.75 min, 40–99% over 0.1 min, and 99% for 5.45 min at a flow rate of 300 nL/min).

### 2.8 Data Analysis

The RAW files were converted to the mzML format using the MSConverter software. For label free data analysis, DIA-NN version 1.8.2 beta^32^ was used for DIA-experiments considering N-terminal acetylation (+42.0106 Da) and methionine oxidation (+15.9949 Da) as variable modifications. Cysteine carbamidomethylation (+57.0214 Da) was applied as a fixed modification for general analyses. Trypsin/P was selected as the digestion enzyme, allowing one missed cleavage. The maximum number of variable modifications was set to two for DIA-based analyses. Peptide length was restricted to 7–35 amino acids, and precursor charge states were set from +2 to +5. For DIA experiments, a scan range of 430–650 *m*/*z* was selected. For cellular proteomics, the *Mus musculus* proteome (UniProtKB, canonical sequences only) was used. Statistical analyses were conducted in R (version 4.5.0) via custom-written scripts.

For the analysis of proteins present in the secretome, we distinguished those annotated in UniProt with a classic signal sequence (predicted extracellular secretion) from nonsecreted proteins (e.g., those potentially originating from cell lysis or membrane shedding).

To be classified as differentially abundant, proteins had to meet two criteria: an adjusted p-value of <0.05 and a magnitude of change greater than 1.5-fold (|log_2_FC| > 0.58).

For the semispecific searches in the LC-MS/MS spectra of the secretome analysis, DIA-NN version 2.5.1 was used in semispecific search mode with tryptic digestion, allowing one missed cleavage, and employing the same fixed and variable modifications defined for the label-free searches described above, accelerated using the InfinDIA module of DIA-NN. Semispecific peptide cleavage events were analyzed using a custom-written script in R.

### 2.9 Data Availability

All mass spectrometry proteomics data have been deposited to the ProteomeXchange Consortium (http://proteomecentral.proteomexchange.org), via the PRIDE partner repository (https://www.ebi.ac.uk/pride/) with project accession PXD079314.

## 3 Results and Discussion

### 3.1 Analysis of the Effects of Adult and Newborn *B. jararaca* Venoms on the Viability of SV40 MES13 Cells

To evaluate the ontogenetic shifts in *B. jararaca* venom toxicity, this study utilized the SV40 MES13 cell line to simulate the murine glomerular response, characterizing changes in both the cellular proteome and the secretome. Accumulated evidence shows that the renal tissue is impacted by systemic effects in the process of envenomation by snakes of the *Bothrops* genus, which if not treated properly can progress to renal failure and death. Kidney damage is driven by direct venom toxicity and indirect, secondary effects. Specifically, venom proteases generate hydrolysis products that overload the glomerular filtration system, while the release of heme from hemolyzed red blood cells intensifies inflammatory and oxidative damage.^33–36^ Thus, this study utilized a glomerular cell lineage to model the venom-induced nephrotoxicity. While ontogenetic shifts in *Bothrops* venom composition are well documented, their specific toxic effects remain poorly understood. This model is therefore essential for characterizing the effects of both the newborn and adult specimens.

The most significant qualitative change in *B. jararaca* venom occurs within the SVMP family, the primary toxins responsible for hemorrhage and tissue damage.^24,37,38^ Newborn venom is characterized by an abundance of P-III-class SVMPs, which are large, complex proteins with potent systemic effects and high procoagulant activity. In contrast, while adult venom still contains P-III SVMPs, it shifts toward P-I class SVMPs, which are smaller molecules composed only of the catalytic domain and typically induce localized hemorrhage and tissue destruction. Beyond the variation in SVMPs, the ontogenetic shift in *B. jararaca* venom is marked by higher abundance of L-amino acid oxidases, C-type lectins, and serine proteinases in the adult venom, optimized for inducing systemic coagulopathy and tissue necrosis in mammalian prey.^25^

Initial assessments of venom activity revealed that the adult venom was significantly more cytotoxic as evidenced by the lower metabolic activity observed in the MTT cell metabolic-viability assay (Figure 1). Indeed, across all tested concentrations (0.25 to 4.0 μg/mL), the newborn *B. jararaca* venom exhibited significantly lower cytotoxicity compared to adult venom, with cell viability remaining markedly higher in the newborn venom-treated cells. Moreover, at the highest venom concentration (4 μg/mL), the cells lost more than 30% of viability when treated with the adult venom while the newborn venom induced a 15% reduction in viability, within 2 h. The lower cytotoxicity observed in newborn venom may reflect the dietary habits of juvenile *B. jararaca*. Since the SV40 MES13 cell line is murine-derived, its relative resistance may stem from the fact that mammals are not typical prey for these snakes in their early life stages.

**Figure 1:**
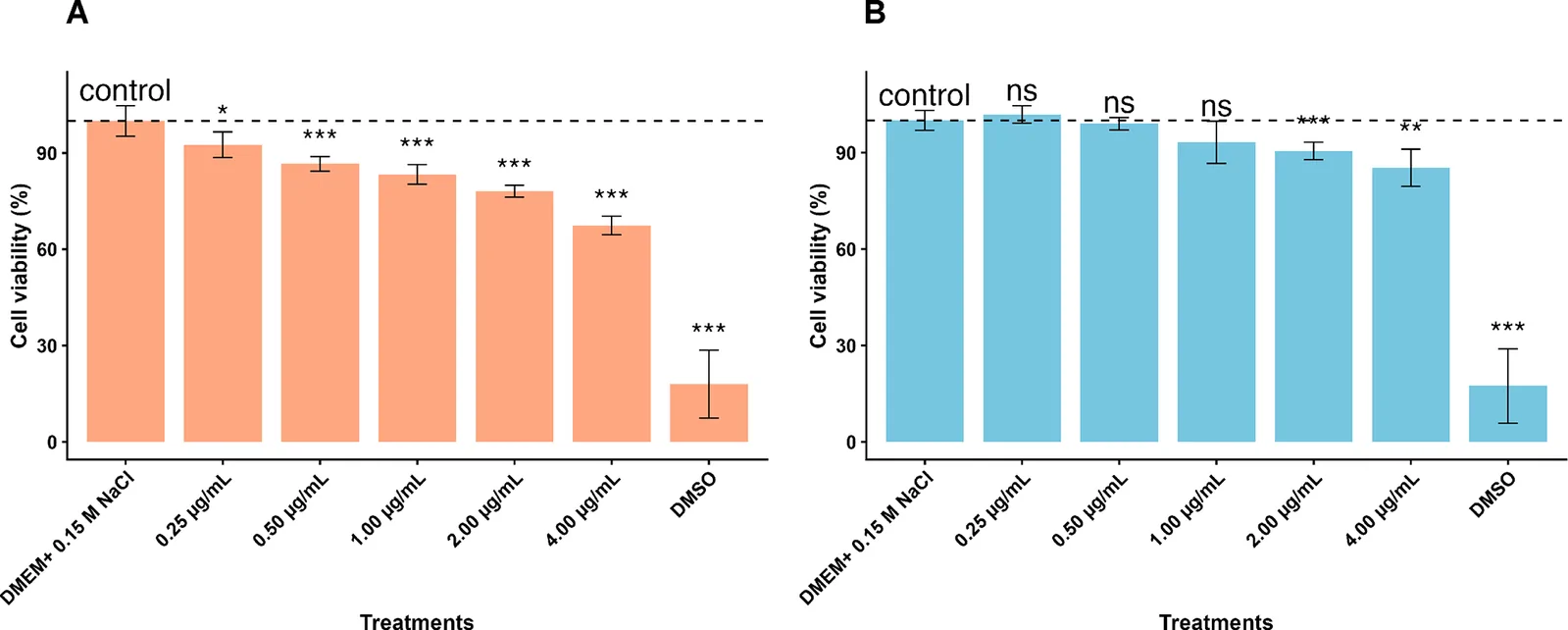
SV40 MES13 cell metabolic-viability assessment following treatment with *B. jararaca* adult and newborn venom. Cells were incubated for 2 h with varying concentrations (0.25, 0.5, 1.0, 2.0, and 4.0 μg/mL) of venom from adult (A) or newborn (B) specimens. Negative control cells (100% viability) were cultured in DMEM (without FBS or phenol red) supplemented with 0.15 M NaCl, while 10% DMSO served as a positive control for cell death. Data are expressed as mean ± standard deviation (SD) of six replicates. Asterisks indicate statistically significant differences (*p* < 0.05), and “ns” indicates nonsignificance, as determined by *t* test with family wise error correction for multiple comparisons.

### 3.2 Adult and Newborn *B. jararaca* Venoms Trigger Distinct Proteomic Alterations in SV40 MES13 Cell Content

For proteomic analysis of the effects of adult and newborn venoms on the cells, increasing venom concentrations (0.25, 0.5, 1.0, 2.0, and 4.0 μg/mL) or 150 mM NaCl (control) were employed. Incubations were carried out in triplicate and each of these six samples was subjected to analysis of the cell content in triplicate, resulting in a total of 108 LC-MS/MS measurements ().

Initially, all samples were converted to mzML format and analyzed using DIA-NN, as described in the Materials and Methods. Preliminary analyses indicated that removing specific samples would enhance the reliability of protein quantification via the *limma* linear modeling approach. Given the robust sampling effort in the study, this adjustment was made without compromising the integrity of the experimental design and statistical analysis. For instance, comparison of chromatographic elution profiles from cells incubated with adult () versus newborn () *B. jararaca* venom revealed that certain samples exhibited suboptimal quality, differing markedly from replicates processed under identical conditions. These chromatographic discrepancies were also reflected in the number of protein identifications across samples (), with inconsistencies ranging from 3,302 to 7,236 identified proteins. We also assessed the distribution of intensity values (), as it provides insights into precursor detection, informs the choice of normalization strategy, and reflects the overall quality of the data sets. In addition, the number of missing values in the matrix generated by the neural network model substantially contributed to the intrinsic variability among samples, thereby reducing analytical precision and impairing the ability to determine whether the observed variation originated from biological differences between experimental conditions or from variability within the same statistical group. Representative examples are seen in the samples of control (0.15 M NaCl) in the group ‘adult venom’ and the 2.0 μg/mL condition in the group ‘newborn venom’. In both cases, protein intensity variation within the experimental group exceeded the 20% threshold considered acceptable for proteomic analysis, as indicated by the dotted lines in . Consequently, all samples that failed to meet these quality criteria were excluded from the final analysis to ensure the integrity of the protein quantification ().

Following the exclusion of suboptimal samples, a total of 8,100 unique proteins were identified across all adult and newborn venom treatments. Of these, over 90% were quantified with a coefficient of variation (CV) below 20%, with the majority of proteins being common to both venom treatments (Figure 2A–D). Notably, Partial Least Squares-Discriminant Analysis (PLS-DA) of the cellular proteome revealed clearer separation between groups treated with adult venom compared to those treated with newborn venom. The adult venom model accounted for 35% of the total variance, whereas the newborn venom model explained only 24% (Figure 2E,F). Furthermore, in the case of adult venom, there was better separation of the groups that received lower concentrations of venom, clustering close to the control groups that was not exposed to the venom, separating the conditions that received 1.0, 2.0, and 4.0 μg/mL of *B. jararaca* venom, possibly due to the more severe changes promoted in the proteome of the cell line used in this study (Figure 2E). In addition, the adult venom profiles exhibited dose-dependent separation. Lower concentrations clustered with the control group, whereas the 1.0, 2.0, and 4.0 μg/mL treatments formed discrete clusters, suggesting that these higher doses promoted more severe proteomic changes in the cells (Figure 2E). In the case of newborn venom, the PLS-DA suggests that the treatment had a negligible effect on the sample profile at low concentrations (0.25–2.0 μg/mL) but triggered a significant shift in the proteomic profile at 4.0 μg/mL.

**Figure 2:**
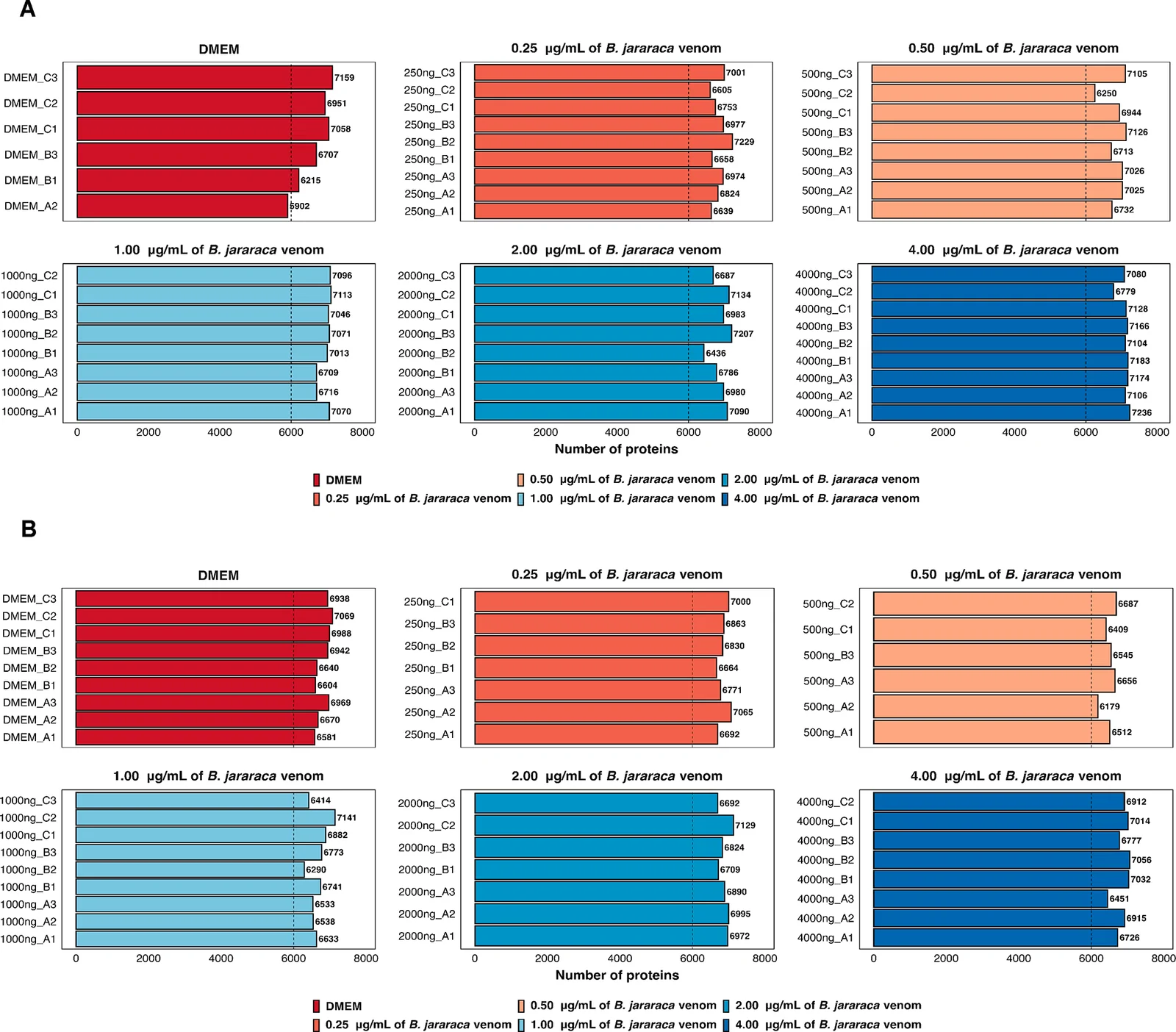

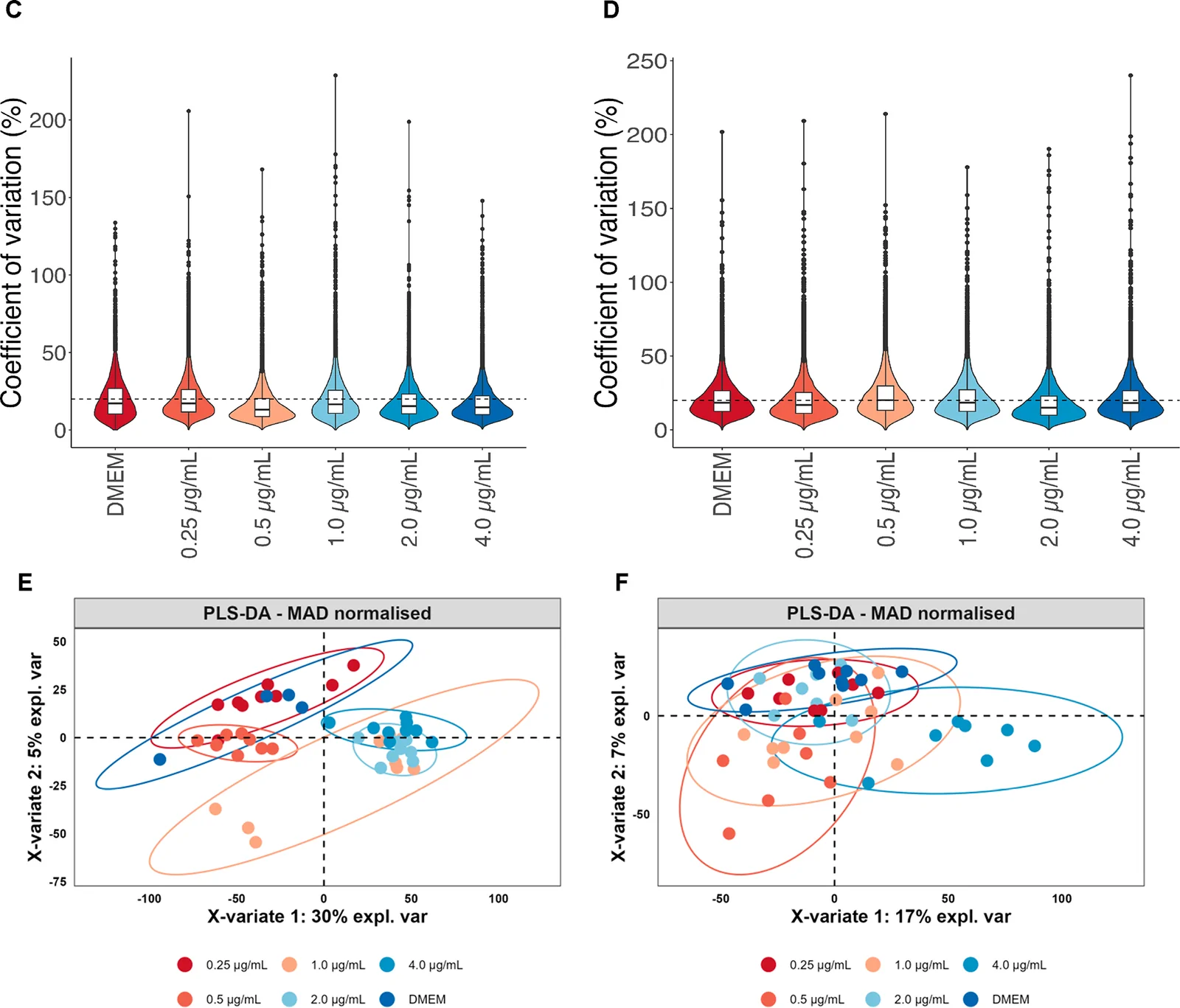
Label-free proteomic analysis of the protein content of SV40 MES13 cells incubated with adult or newborn *B. jararaca* venom for 2 h. Number of proteins identified in the cellular protein content across samples exposed to adult (A) or newborn (B) venom. Bars represent individual sample replicates. Treatment concentrations shown above each plot in μg/mL correspond to the *y*-axis sample prefixes expressed in ng/mL (e.g., 500 ng = 0.50 μg/mL). Replicate codes combine the biological replicate letter (A, B, or C) and technical replicate number (1, 2, or 3). Coefficient of variation between samples of adult (C) and newborn (D) venom. The dotted line indicates a variation less than or equal to 20%, indicating that the variation between proteins within the same group does not influence the biological variation between groups. Partial least-squares-discriminant analysis of cell content samples incubated with increased concentrations of adult (E) and newborn (F) venom.

Applying *limma* statistics^39^ and using the least-squares regression model, it was possible to quantify more than 7,300 proteins in terms of relative differential abundance in cells incubated with different concentrations of *B. jararaca* venom for 2 h (). Volcano plots revealed a dose-dependent increase in the number of differentially abundant proteins. Specifically, adult venom treatment significantly altered the proteome, with 544 and 617 proteins increasing in abundance at 2.0 and 4.0 μg/mL, respectively (Figure 3A). In contrast, newborn venom had a much smaller impact on the cells, promoting the increase of abundance of respectively only 2 and 65 proteins at the same concentrations (Figure 3B).

**Figure 3:**
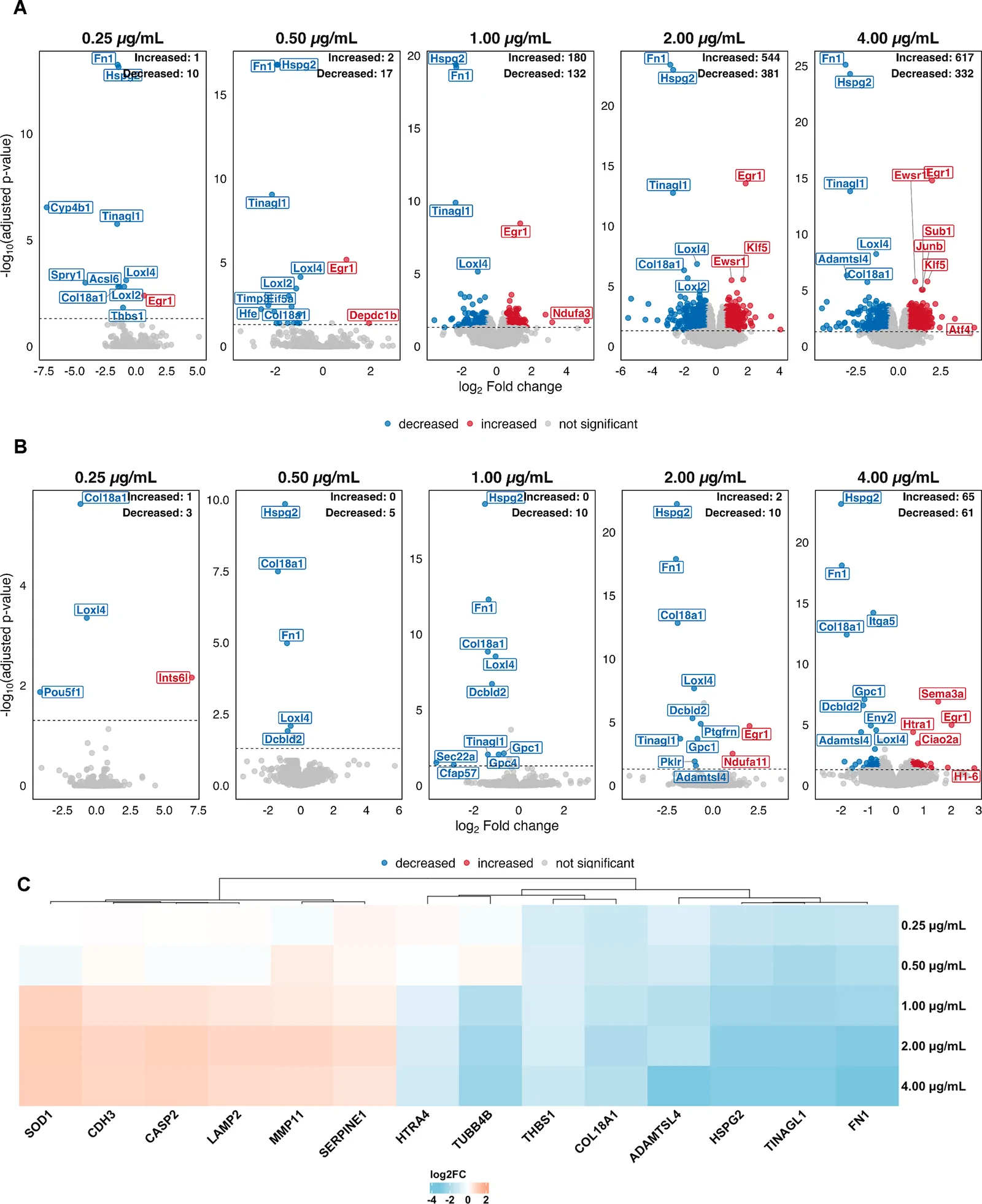

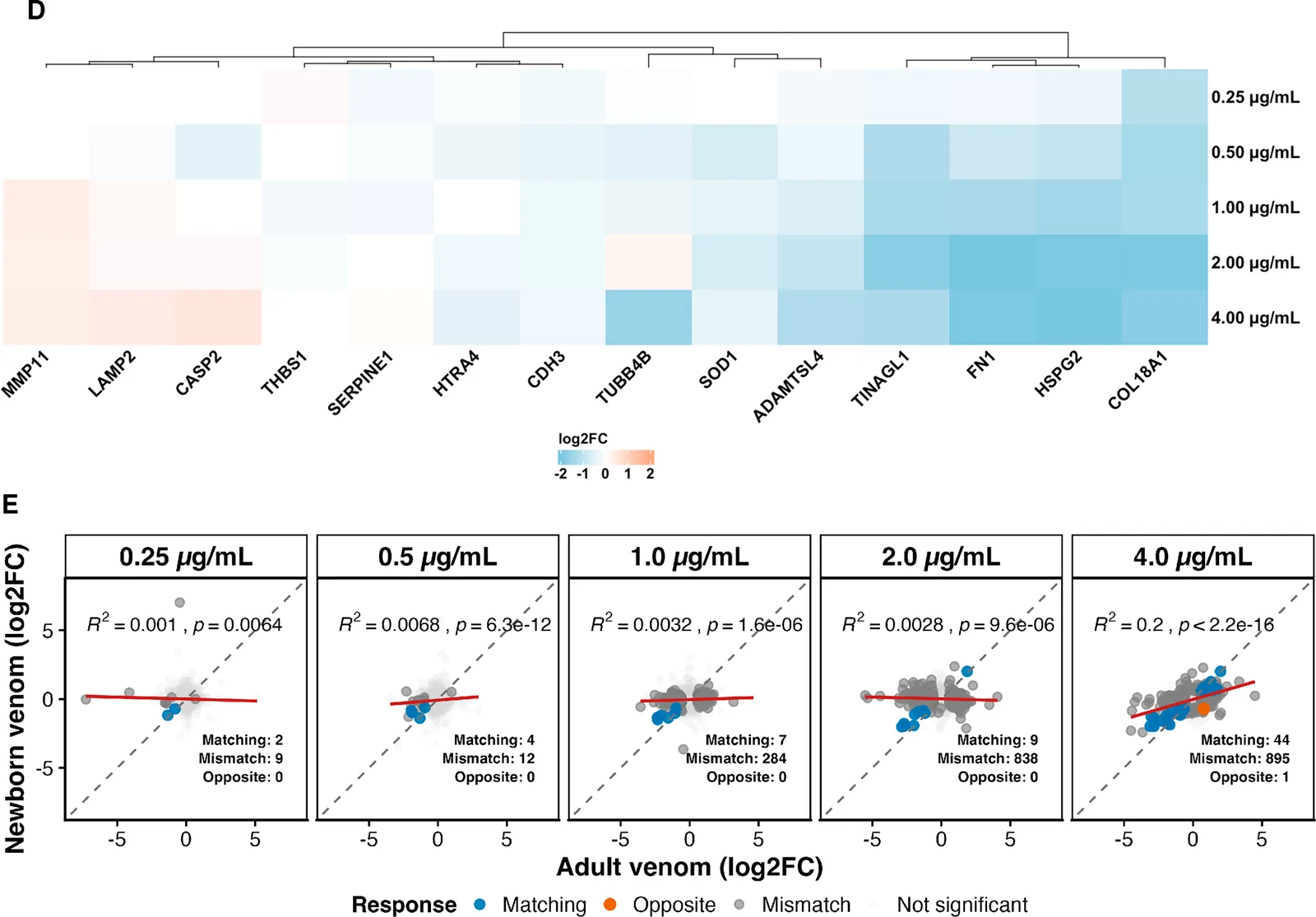
Label-free proteomic analysis of the protein content of SV40 MES13 cells incubated with *B. jararaca* adult or newborn venom for 2 h. Volcano plots showing the number of differentially identified proteins in the cell content by mass spectrometry, after analysis using a linear model (limma), comparing different treatments (0.25, 0.5, 1.0, 2.0, and 4.0 μg/mL) of *B. jararaca* adult (A) and newborn (B) venom relative to the control (DMEM + 0.15 m NaCl). Heat maps comparing the log_2_ fold change of differentially abundant proteins identified in the cell content of samples incubated with increasing concentrations of *B. jararaca* adult (C) and newborn (D) venom. (E) Pearson’s correlation between log_2_FC newborn venom in *y* axis and adult venom in *x* axis. Proteins were classified as “matching” when the direction of the change agreed (increased, decreased in both comparisons), “opposite” when direction of the change disagreed (increased in one and decreased in the other), and “mismatch” when the proteins were significantly changed in one comparison but not in the other. Numbers of proteins are depicted at the bottom of each panel with the protein response.

Figure 3C,D show heatmaps illustrating the differential impact of adult and newborn venoms on a panel of 14 selected proteins across the venom concentration gradient (0.25 to 4.00 μg/mL). The variations in protein abundance reflect the active response of SV40 MES13 cells to the toxic insult and illustrate how their internal machinery was modulated by the activity of venom toxins. Interestingly, protein abundance variation, expressed as log_2_FC, revealed distinct patterns in venom-induced cellular response.

Considering the cell viability data, where only the 4 μg/mL dose caused a higher significant decrease in survival in both venoms (Figure 1), the abundance variations of 14 selected important proteins across the venom concentration gradient allowed for their classification into functional groups. Overall, the analysis revealed a silent phase of damage where the cells remained viable, yet their molecular machinery was already compromised.

The first group is composed by up-regulated proteins involved in survival response. Superoxide Dismutase 1 (SOD1), increased even at mid-doses (1.0 μg/mL), suggesting that the adult venom generated reactive oxygen species. The cells were alive because SOD1 was likely successfully buffering the oxidative stress up to 4.0 μg/mL, where the system likely became overwhelmed. Interestingly, caspase 2 (CASP2), a pro-apoptotic marker, rose before the cells actually died. It suggests the cells were primed for death at 1.0–2.0 μg/mL of adult venom, but the actual execution of apoptosis only hit the 30% mark at the highest dose (4.0 μg/mL). Lysosome-associated membrane protein 2 (LAMP2), a protein involved in autophagy, showed higher abundance, suggesting the cells were trying to digest damaged proteins or organelles caused by the adult venom to stay alive.

The second group comprises increased proteins associated with tissue remodeling, indicating that adult venom toxins induced a phenotypic transition toward a fibrotic state. Specifically, the elevation of serpine 1 (plasminogen activator inhibitor-1), a recognized biomarker of renal pathology, suggests that the venom promoted the inhibition of the proteolytic turnover of the extracellular matrix (ECM). *In vivo*, this impairment of matrix clearance would culminate in glomerulosclerosis. In parallel, the increase in stromelysin-3 (MMP11) facilitated the proteolysis of ECM components, correlating with the diminished abundance of structural proteins. Furthermore, the rise in cadherin-3 (CDH-3), a mediator of cell–cell adhesion, likely functions as a compensatory mechanism to maintain intercellular cohesion as the underlying basement membrane undergoes enzymatic degradation.

The third group is composed of proteins identified with different degrees of reduced abundance essentially in all adult venom concentrations tested. The most dramatic decreases were observed in structural and adhesion proteins, particularly fibronectin 1 (FN1). As a critical mediator of cell-matrix interactions, the enzymatic degradation of FN1 leads to disruption of the physical tethering of cells to the extracellular matrix, potentially triggering anoikis. The reduction of perlecan and collagen XIII indicates an impact on the cells’ immediate microenvironment, likely destabilizing the structural integrity of the cultured monolayer. As tubulin figures in the cytoskeleton, its decrease means the cells are physically collapsing. ADAMTS-like protein 4 (ADAMTSL4) is essential for microfibril assembly and its decline underscores the venom targeted disruption of the specialized glomerular architecture. Similarly, high-temperature requirement factor A4 (HTRA4), a protease critical for cell signaling, exhibited a distinct downward shift at higher concentrations, suggesting a late-stage failure in cellular communication.

In light of the *in vitro* nature of these findings, the comparative data suggests that the adult venom induced a more profound disruption of the cultured cellular environment than the newborn venom. With the adult venom yielding a 30% reduction in cell viability, which is twice the 15% observed with newborn venom, it is evident that the adult venom exerted a more aggressive proteolytic demand on the cell culture. This was likely driven by a higher concentration of specific SVMPs in the adult venom that forced an enhanced MMP11/SERPINE1 response within the culture. Specifically, the adult venom is more efficient at striking the basement membrane architecture. The accelerated degradation of ECM components such as FN1 and collagen alpha-1­(XVIII) chain (COL18A1) likely led to comprehensive substrate deadhesion, ultimately triggering detachment-induced apoptosis. While the newborn venom exerted lower oxidative stress as evidenced by a more modest SOD1 response, the adult venom rapidly pushed the culture beyond its oxidative threshold. Essentially, these data revealed a distinct sequence of decline possibly with a functional death occurring at 1.0 μg/mL as the cells lose the structural proteins required for adherence, preceding the physical death and partial loss of viability observed at 4.0 μg/mL.

*Bothrops* venoms are rich in proteases that disrupt homeostatic balance by degrading basement membrane proteins and components essential for cell-matrix and cell–cell adhesion.^40^ The proteomic alterations observed in this study, specifically those involving cytoskeletal organization and cellular adhesion, complement our cell viability data to suggest the occurrence of anoikis. This form of programmed cell death is likely triggered by the degradation of extracellular matrix components by SVMPs.^41–45^ Furthermore, PLA2s may act synergistically to promote cell detachment, further explaining the reduced viability observed in the MTT assays.^46,47^ However, this phenomenon may be significantly more complex, involving an orchestration of host tissue proteases and various signaling pathways activated during envenomation. Furthermore, Pearson correlation analysis (Figure 3E) highlighted the distinct proteomic profiles of cells exposed to adult versus newborn venoms, particularly at lower concentrations, where the proteins showing altered abundance were largely distinct between the two conditions. The analysis suggests that most of the significant proteins, according to the log_2_FC and adjusted P-value criteria, exhibited increased or decreased abundance exclusively in response to one of the two venoms (mismatch), indicating that adult and newborn venoms induce distinct cellular responses. As the venom concentration increased, as observed at 4 μg/mL, the number of proteins exhibiting similar abundance patterns (matching) between the two treatments also increased. This finding suggests that both conditions activated shared cellular response pathways. These common responses to venom-induced injury likely involve canonical cell death and stress-response pathways.

### 3.3 Adult and Newborn *B. jararaca* Venoms Induce Divergent Changes in the SV40 MES13 Cell Secretome

To complement the cellular analysis, the secretory profile was examined using the same experimental design applied for the analysis of the cell content. By maintaining a triplicate structure for both the incubations and the subsequent mass spectrometry runs, the resulting data set comprised 108 individual measurements ().

A preliminary analysis using all secretome samples was performed to investigate the quality of the LC-MS/MS acquisitions. As previously applied to the cellular content data set, a series of quality control analyses were conducted to increase the reliability and accuracy of protein quantification using the linear model. In the secretome data set, for both conditions (cells incubated with adult or newborn venom), the number of missing values (proteins) () was higher than that observed with the cellular content samples, which was expected given the dynamic nature of the extracellular environment, where proteins may or may not be secreted and in varying amounts depending on the stimuli to which the cell culture was exposed. An essential consideration is that of the continuous exposure of cells to venom proteases, which, according to their substrate specificity, cleave proteins present in membranes and the extracellular matrix. In some cases, this process may lead to cell death and release of cytoplasmic contents, drastically altering the proteome present in the conditioned medium (secretome), as well as the variation in the number of identified proteins (), ranging from 1,095 to 4,939 proteins, and the acquisition intensities (). Collectively, these variables increased the intragroup variance (), which may have compromised the accuracy of protein quantification. To ensure data quality, samples with high proportions of missing values and elevated coefficients of variation were excluded from subsequent analyses ().

Following this filtration process, analysis of the secretome identified over 5,500 unique proteins across all samples (Figure 4A,B). Approximately 25% of these proteins contained a signal peptide, indicating that they were destined for the classical secretory pathway. Incubation with adult *B. jararaca* venom resulted in a significant expansion of the secretome content with an increase of nearly 300 unique proteins. This enhanced protein diversity likely stems from the experimental conditions, where continuous exposure to a protease-rich venom with broad substrate specificities promoted the cleavage of various cell-surface and extracellular matrix components. Given the high variability in the dynamics of the proteins present in the conditioned medium and the nature of the experiment, the secretome samples exhibited coefficients of variation slightly higher than those observed for the cellular content samples (Figure 4C,D).

**Figure 4:**
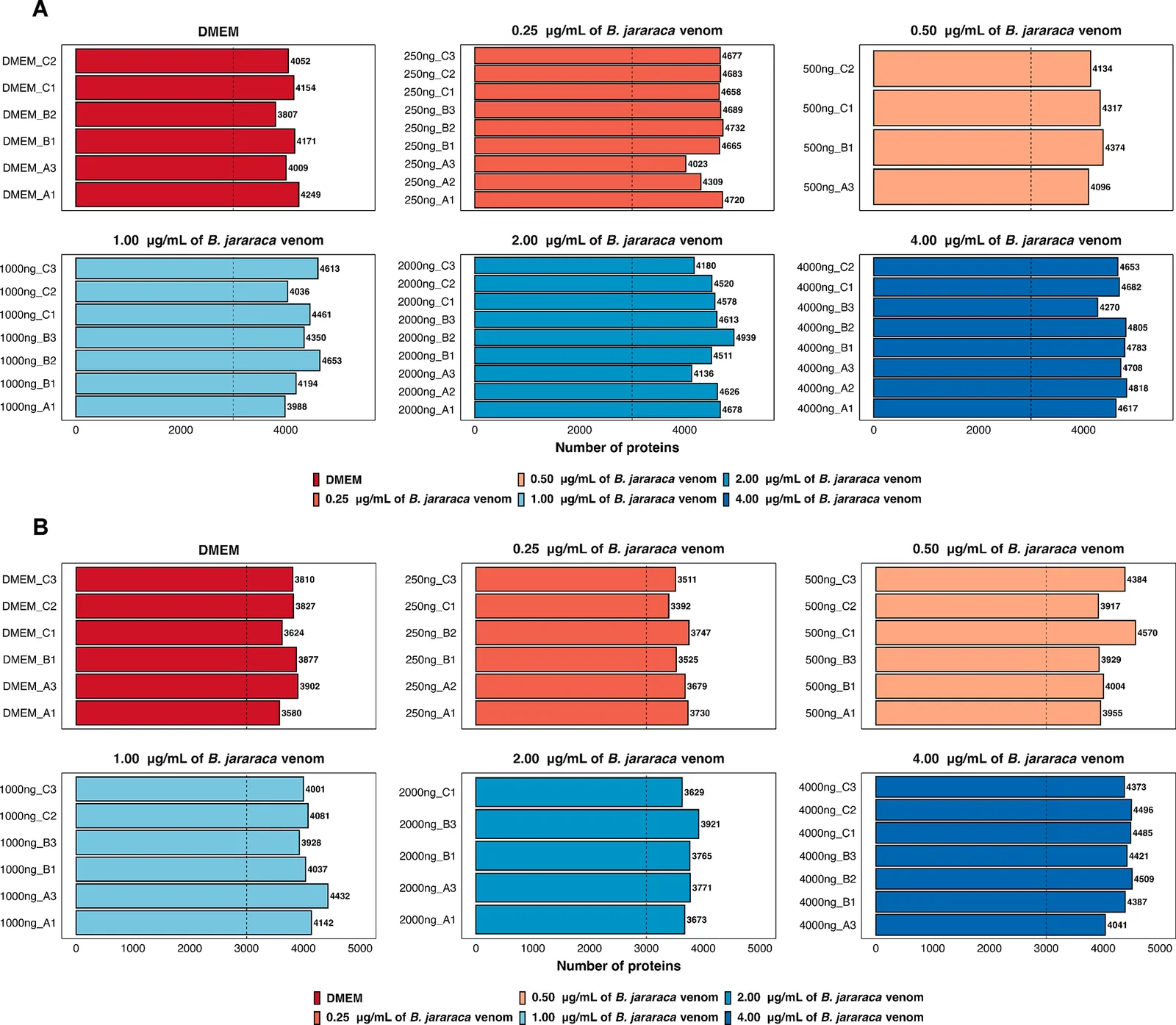

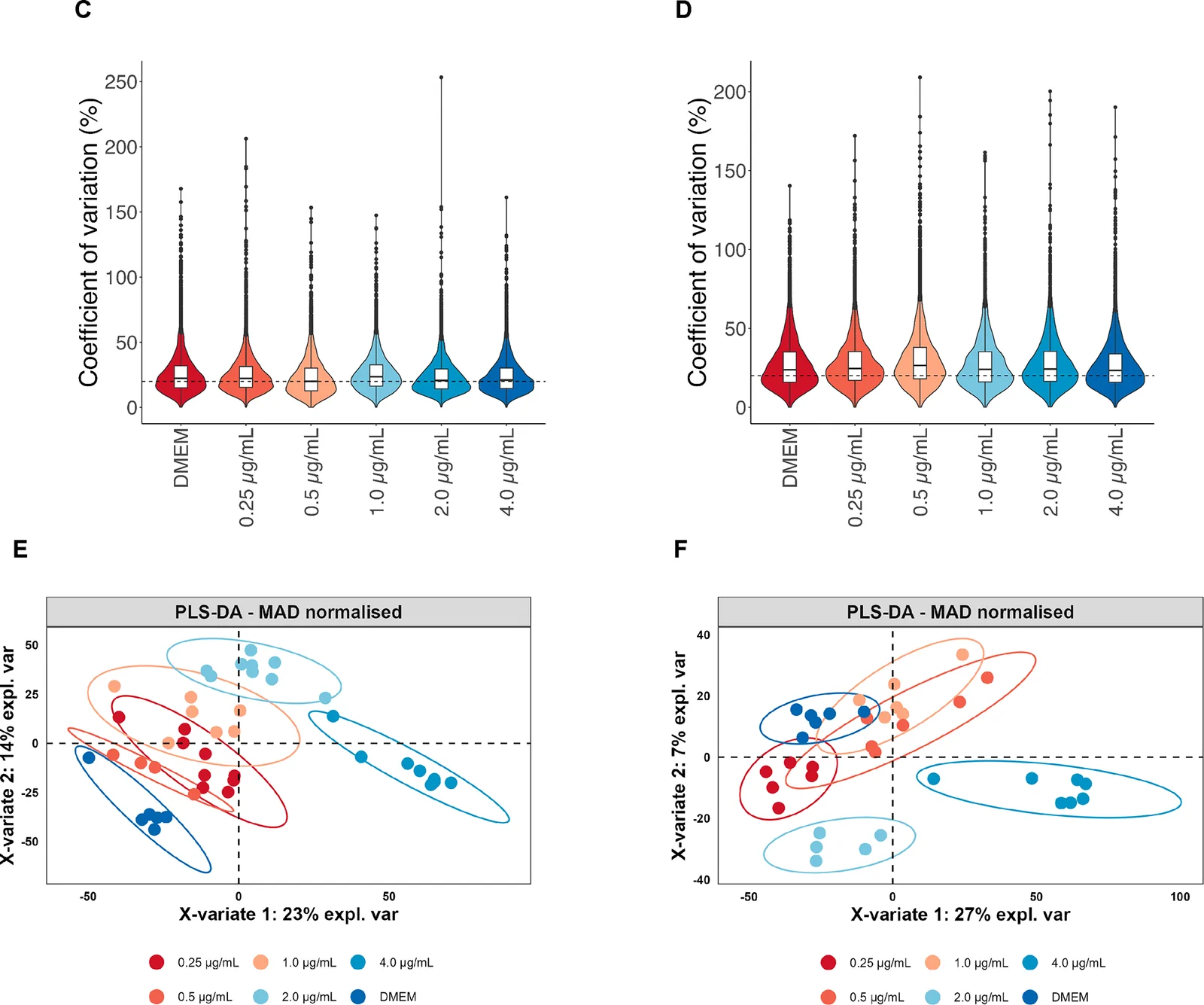
Label-free proteomic analysis of the secretome of SV40 MES13 cells incubated with *B. jararaca* adult or newborn venom for 2 h. Number of proteins identified in the cellular secretome across samples in the adult (A) and newborn (B) venom. Bars represent individual sample replicates. Treatment concentrations shown above each plot in μg/mL correspond to the *y*-axis sample prefixes expressed in ng/mL (e.g., 500 ng = 0.50 μg/mL). Replicate codes combine the biological replicate letter (A, B, or C) and technical replicate number (1, 2, or 3). Coefficient of variation between samples of adult (C) and newborn (D) venom. The dotted line indicates a variation less than or equal to 20%, indicating that the variation between proteins within the same group does not influence the biological variation between groups. Partial least-squares-discriminant analysis of cell secretome samples incubated with increased concentrations of adult (E) and newborn (F) venom.

Consistent with the results for the cellular proteome, the secretome of cells incubated with adult venom provided enhanced group discrimination compared to those incubated with newborn venom. The PLS-DA of secretome of cell incubated with adult venom accounted for 37% of the explained variance, compared to 34% for the newborn venom. Notably, the secretome data sets yielded a more pronounced separation of experimental groups than the corresponding cellular content samples (Figure 4E,F). Furthermore, secretome analysis revealed a dose-dependent clustering pattern. While lower venom concentrations grouped closely with the control, treatments of 1.0, 2.0, and 4.0 μg/mL exhibited distinct separation. This divergence suggests that higher concentrations induce more profound alterations in the cellular secretome.

Statistical analysis using limma allowed for the robust quantification of a significant subset of proteins identified in the secretome following venom exposure (Figure 5A,B and ). Because the experiment exposed cell cultures to protease-rich venoms, the protein composition of the conditioned medium was expected to change significantly. Indeed, the identified proteins comprised naturally secreted proteins, whose release was either enhanced or unaffected by the venom, as well as proteins derived from membrane cleavage and cell lysis. As the venom concentration increased, we observed a gradual increase in proteins lacking secretion-targeting signal peptides, which are not categorically associated with the canonical cellular secretion pathway. This effect was particularly pronounced for the adult snake venom, where even the lowest concentrations (0.25 and 0.5 μg/mL) significantly altered nonsecreted protein levels relative to the control group (*limma* linear model). Conversely, newborn snake venom elicited a gradual, dose-dependent increase in these proteins, peaking at the highest concentration of 4 μg/mL. For the subset of secreted proteins featuring annotated UniProt signal peptides, the treatment with either adult or newborn venom at high concentrations (2 and 4 μg/mL) elicited the most significant differential abundance compared to control group levels. Under these conditions, 77 and 86 proteins were found to be increased following exposure to adult venom, whereas 47 and 106 increased proteins were observed for the newborn venom, respectively. Although the newborn venom generated a higher number of increased proteins annotated as secreted, the adult venom promoted a substantial increase in the number of intracellular or membrane proteins. These reductions were most likely associated with protein degradation occurring directly in the conditioned medium, mediated either by venom proteases or by cellular proteases activated in response to the venoms’ toxic effects. In this context, treatment of cells with 2 and 4 μg/mL of adult venom resulted in a decrease of 56 and 51 secreted proteins, respectively, compared with the 9 and 24 observed for newborn venom.

**Figure 5:**
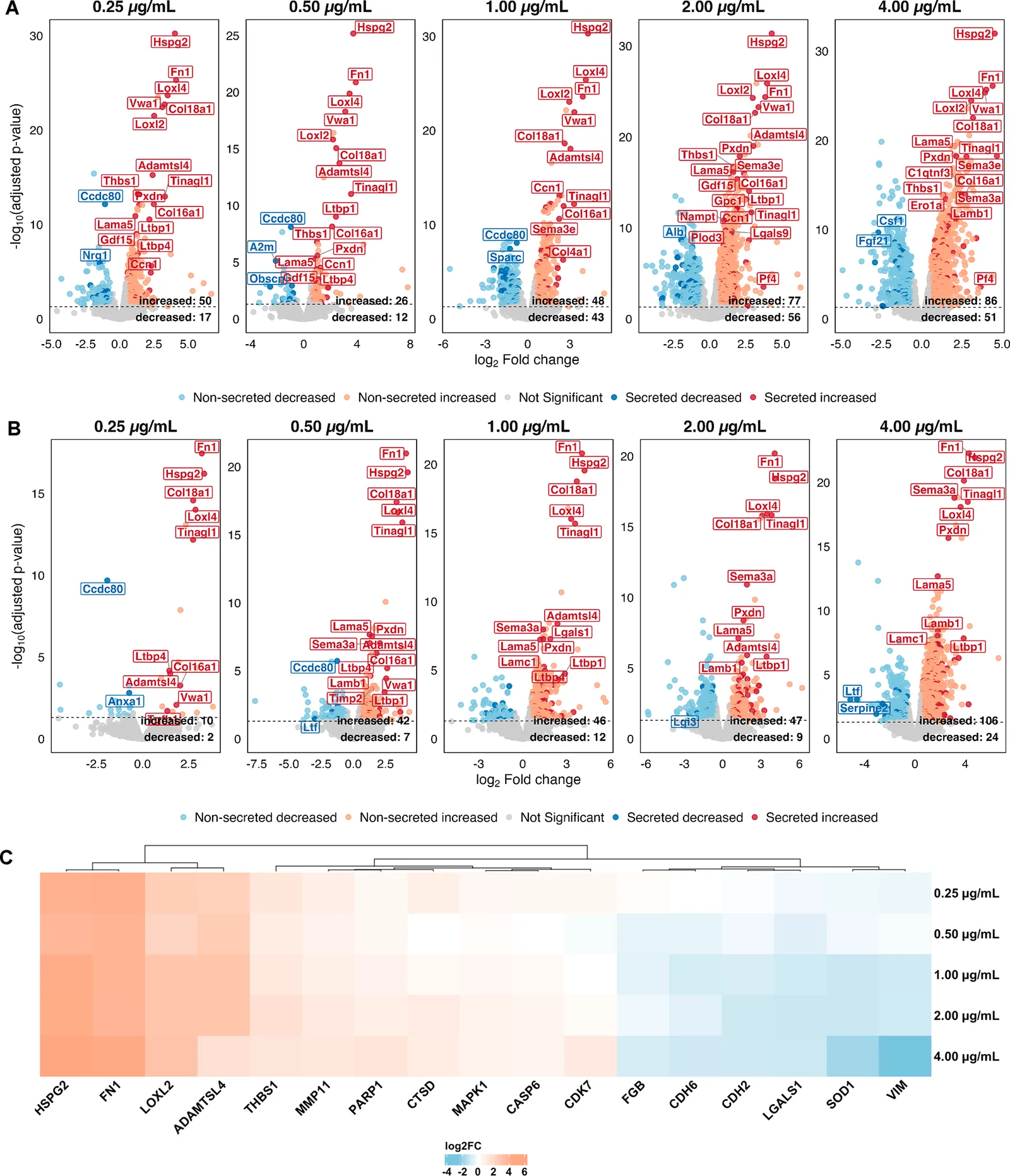

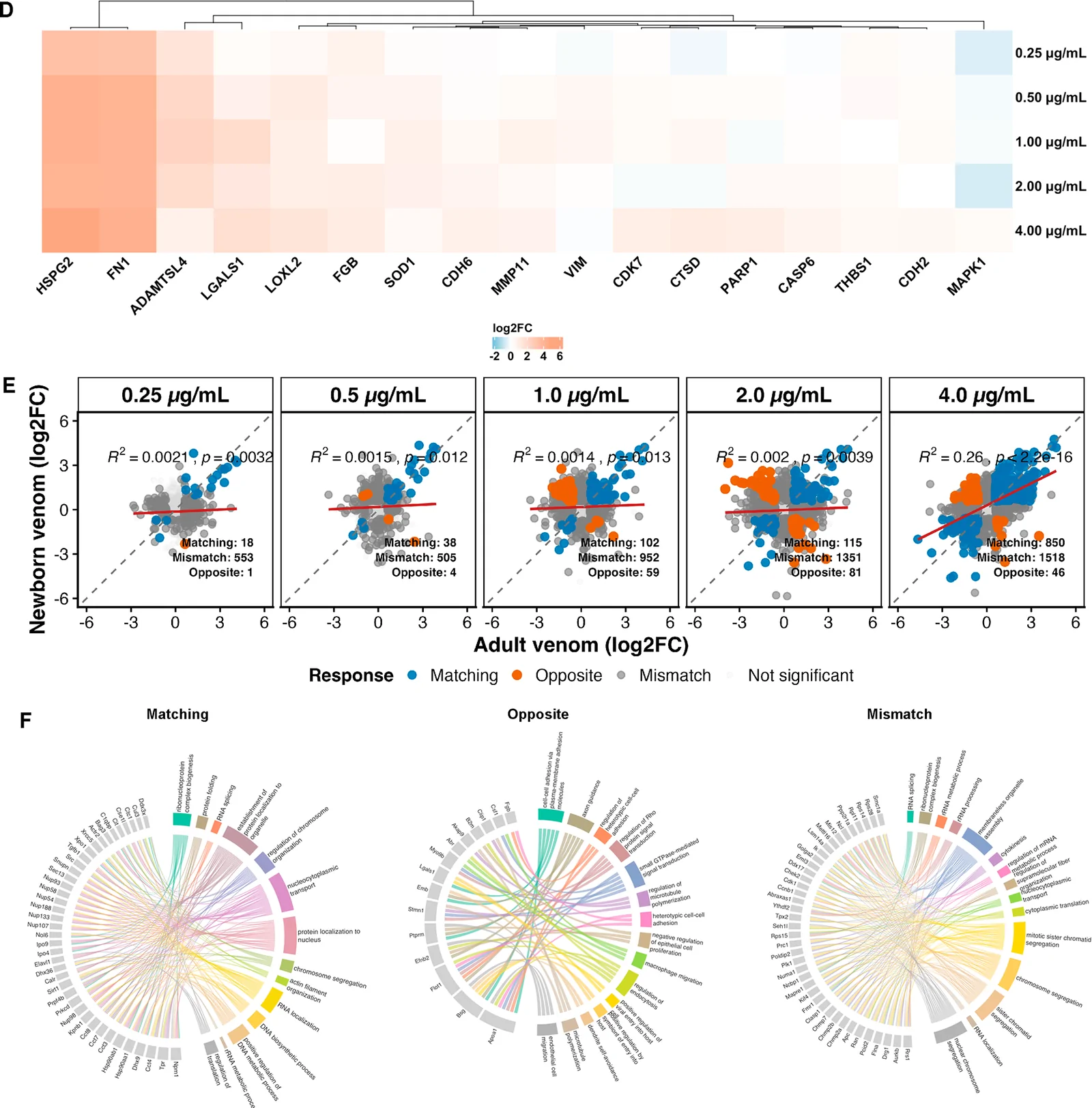
Label-free proteomic analysis of the secretome of SV40 MES13 cells incubated with *B. jararaca* adult or newborn venom for 2 h. Volcano plots showing the number of differentially identified proteins in the secretome by mass spectrometry, after analysis using a linear model (limma), comparing different treatments (0.25, 0.5, 1.0, 2.0, and 4.0 μg/mL) of *B. jararaca* adult (A) and newborn (B) venom relative to the control (DMEM + 0.15 m NaCl). Heat maps comparing the log_2_ fold change of differentially abundant proteins identified in the secretome of samples incubated with increasing concentrations of *B. jararaca* adult (C) and newborn (D) venom. (E) Pearson’s correlation between log_2_FC newborn venom in *y* axis and adult venom in *x* axis. Proteins were classified as “matching” when the direction of the change agreed (increased, decreased in both comparisons), “opposite” when direction of the change disagreed (increased in one and decreased in the other), and “mismatch” when the proteins were significantly changed in one comparison but not significant in the other. Numbers of proteins are depicted at the bottom of each panel with the protein response. (F) Chord diagrams showing the enriched biological processes based on proteins that exhibited similar responses between the venoms of adult and newborn snakes (matching), opposite responses (opposite), or changes in abundance on only one side (mismatch), as determined by the previous analysis. The 15 most significant biological processes associated with at least two proteins were selected, using a p-value ≤ 0.05 and controlling the false discovery rate (FDR) according to the Benjamini–Hochberg method.

Considering the secreted proteins, we selected 17 proteins based on their biological functions and the magnitude of their abundance changes (Figure 5C,D). Among them, heparan sulfate proteoglycan 2 (HSPG2) and FN1 showed a marked increase in abundance in the conditioned medium following treatment with both venoms, with log2FC values of greater than 4 at the highest concentrations tested. These proteins consistently ranked among the most differentially abundant ones identified in the venom-treated versus control comparisons following *limma* analysis and the application of log2FC and adjusted p-value thresholds.

While their upregulation is traditionally associated with *in vivo* renal pathologies like fibrosis, where excessive extracellular matrix deposition drives tissue damage, their marked increase in this *in vitro* model suggests that they serve as early cellular markers of venom-induced cytotoxicity and injury.^48,49^ Further reflecting this shift toward a pro-fibrotic phenotype and active extracellular matrix remodeling in culture, lysyl oxidase homolog 2 (LOXL2), MMP11, and ADAMTSL4 also exhibited increased abundance across both venom-treated experimental conditions. LOXL2 catalyzes the cross-linking of collagen and elastin within the extracellular matrix through the oxidative deamination of lysine residues, and its dysregulation is strongly associated with tissue fibrosis.^50^ Similarly, MMP11 is a zinc-dependent metalloprotease secreted as a zymogen that requires proteolytic cleavage of its propeptide for activation. While its role in extracellular matrix degradation and remodeling is classically associated *in vivo* with progressive renal injury, such as glomerulonephritis,^51^ its upregulation in this cell culture model suggests that snake venom exposure actively triggers matrix-remodeling pathways at the cellular level, potentially via the direct activation of host proteases or by the action of venom toxins. Finally, ADAMTSL4 is involved in the regulation of extracellular matrix organization, and its aberrant activity has been linked to fibrogenic processes in other organs.^52^

Similarly, another group of proteins involved in cellular signaling and inflammatory response pathways was identified as differentially abundant upon cell treatment with both venoms. The proteins thrombospondin-1 (THBS1), poly [ADP-ribose] polymerase 1 (PARP1), mitogen-activated protein kinase 1 (MAPK1), and cyclin-dependent kinase 7 (CDK7) exhibited dose-dependent variations in abundance in response to both venoms compared with the control treatment after 2 h of incubation. THBS1, a glycoprotein frequently associated with tissue repair and inflammatory processes, was found increased in the secretome of cells incubated with both adult and newborn venoms and has previously been associated with fibrotic and inflammatory processes in renal tissue.^53,54^ PARP1 and CDK7, which are involved in DNA repair, cell cycle regulation and transcriptional control, respectively, were also found upregulated in both treatments, suggesting a cellular effort to preserve homeostasis and maintain genomic integrity, thereby supporting cellular replication and survival.^55,56^ In contrast, MAPK1, a serine/threonine-specific protein kinase, displayed distinct abundance patterns between cells exposed to adult and newborn venoms. In the secretome of cells treated with adult venom, MAPK1 abundance progressively increased with higher venom concentrations. Conversely, in cells exposed to newborn venom, MAPK1 abundance was initially lower than the control and increased only at a concentration of 4 μg/mL of *B. jararaca* venom. The increased abundance of this kinase may be associated with the activation of inflammatory signaling pathways, promoting the production of cytokines such as TNF-α, IL-1, and IL-6, as well as the activation of additional kinases within the MAPK signaling cascade.^57^

Furthermore, increased levels of cysteine and aspartic proteases were identified in the secretome of cells incubated with both venoms, albeit to varying degrees. Among these, caspase-6 (CASP6) and cathepsin D (CTSD) were particularly noteworthy. CASP6 contains a cysteine residue at its catalytic active site as part of its reactive mechanism and is classified as an executioner caspase, acting during the execution phase of apoptosis by the cleavage of structural proteins. In the present study, the progressive increase in CASP6 abundance, particularly at venom concentrations of 2 and 4 μg/mL, suggests the occurrence of apoptotic events accompanied by a partial reduction in cellular viability, consistent with the results obtained from the MTT assays.^58^ CTSD, a lysosomal aspartic protease whose physiological functions are associated with apoptosis and MHC class II antigen processing, was also identified as upregulated under both experimental conditions and has additionally been correlated with renal fibrosis and lysosomal protein degradation processes.

In many cases, this cathepsin is secreted as an inactive zymogen. Its activation relies on the proteolytic processing of its propeptide by other proteases, such as matrix metalloproteinases, which, in turn, can be activated by venom proteases, ultimately driving CTSD proteolytic activity toward its substrates.

Finally, another group of proteins playing crucial roles in the maintenance of cellular structure, including adhesion-related proteins such as galectin-1 (LGALS1), cadherin-2 (CDH2), and cadherin-6 (CDH6), as well as the cytoskeletal protein vimentin (VIM), exhibited distinct differential abundance patterns between cells incubated with adult and newborn venom. In the secretome of cells exposed to adult venom, a clear trend toward reduced abundance relative to the control was observed, which may suggest extensive proteolytic processing or degradation of these structural proteins. In contrast, cells exposed to newborn venom exhibited increased abundance of these proteins in the secretome, possibly reflecting a cellular response to venom-induced stress. LGALS1, which is recognized for its roles in cell-matrix interactions and regulation of acute and chronic inflammatory responses,^59^ was markedly increased, particularly at higher concentrations (1, 2, and 4 μg/mL) of newborn *B. jararaca* venom, whereas reduced abundance was observed in cells treated with adult venom. Similarly, CDH2, CDH6, and VIM, which are also associated with cell adhesion and cytoskeletal organization, displayed abundance profiles comparable to those observed for LGALS1. These findings suggest that adult snake venom possesses a greater capacity to degrade cell adhesion proteins, potentially contributing to the more pronounced loss of cell viability observed under these conditions. The disruption of adhesion sites and cytoskeletal integrity may promote detachment of the cellular monolayer and trigger cell death mechanisms such as anoikis, caspase activation, including CASP6, and autophagy.^60,61^ Interestingly, the differential abundance profile of SOD1 varied markedly between the treatments, highlighting contrasting cellular responses to increasing venom concentrations. Upon exposure to adult venom, it exhibited a sharp, dose-dependent downregulation that stabilized into a robust, sustained increase at doses from 1 to 4 μg/mL. Conversely, under the effect of the newborn venom SOD1 was detected in higher abundance at 0.5 μg/mL and above. This divergence suggests that while the newborn venom triggered a high-dose antioxidant or cellular stress response via SOD1 elevation, the adult venom instead led to a suppression of this protein at high venom concentrations, affecting apoptotic pathways.^62^

Similarly, as observed for the cellular content, the Pearson correlation analysis (Figure 5E) indicated low convergence between the proteins identified in the secretome, particularly at the lower venom concentrations, where the correlation was virtually negligible. As the venom concentration increased, the number of proteins exhibiting similar abundance patterns between the two treatments also increased, accompanied by a modest improvement in the overall correlation, reaching approximately 850 proteins with matching status between the adult and newborn venom treatments at 4 μg/mL. Under this condition, analysis of the biological processes (Figure 5F) associated with these proteins revealed that those classified as matching, i.e., exhibiting similar abundance patterns in response to both venoms, were primarily involved in fundamental cellular processes, including structural maintenance, chromosome segregation, and cell division. In contrast, proteins classified as opposite, corresponding to proteins exhibiting divergent abundance patterns between the two venom treatments, were predominantly associated with cell adhesion, regulation of cellular signaling and defense and cell-matrix adhesion, consistent with the findings presented thus far. Finally, proteins classified as mismatch, indicating proteins altered in response to one venom but not to the other, were mainly associated with RNA processing and cell division.

Additionally, we compared the normalized intensities of a subset of proteins detected in both the cellular content and the secretome. As venom concentration increased, COL18A1, FN1, basement membrane-specific heparan sulfate proteoglycan core protein (HSPG2), LOXL4, and tubulointerstitial nephritis antigen-like protein (TINAGL1) exhibited reduced abundance in the cellular content accompanied by a marked increase in the secretome for both venoms, except for thrombospondin-1 (THBS1), which remained practically stable for the newborn venom (Figure 6A,B).

**Figure 6:**
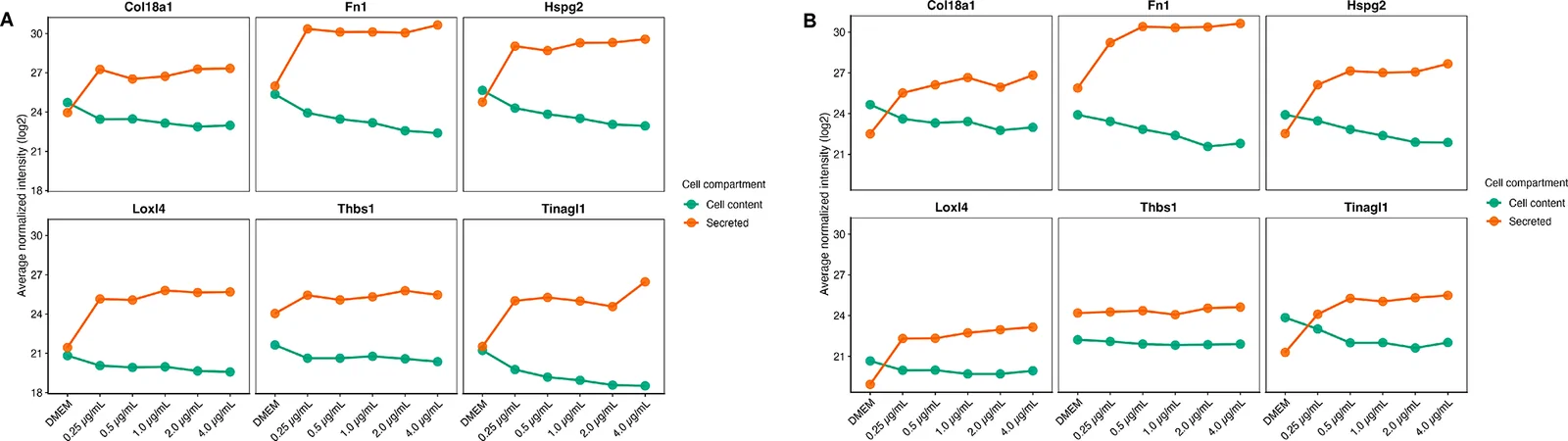
Average intensity normalized by scale of different proteins identified in the cellular content and secretome of cells incubated with adult *B. jararaca* venom for 2 h. The graphs show a tendency of selected proteins (collagen XVIII, Col18a1; fibronectin, Fn1; basement membrane-specific heparan sulfate proteoglycan core protein, Hspg2; lysyl oxidase homologue 4, Loxl4; thrombospondin-1, Thbs1; and tubulointerstitial nephritis antigen-like, Tinagl1) to show decreased intensity in the cellular content and increased intensity in the secretome of cells incubated with adult venom (A) or newborn venom (B) along with the venom concentration range.

For COL18A1, a pronounced increase was detected even at the lowest concentration of adult venom incubated with the cells (0.25 μg/mL), suggesting an immediate cellular response to the insult induced by venom toxins, whereas the newborn venom elicited a more gradual increase, peaking at the highest venom concentration tested (4 μg/mL). FN1 and HSPG2 exhibited similar patterns in both treatments, characterized by an immediate increase under the initial adult venom incubation condition (0.25 μg/mL), whereas the response to newborn venom was more gradual. For LOXL4 and TINAGL1, the differences in normalized intensities were more pronounced when comparing the two venoms, with both treatments exhibiting a similar pattern of increased abundance in the extracellular compartment, accompanied by a reduction in intracellular abundance in cells incubated with adult venom. In contrast, cells exposed to newborn venom exhibited a sharper pattern. Finally, THBS1 followed the same overall pattern as the other proteins, though less prominently, and its normalized intensity remained relatively stable after exposure to newborn venom.

On the other hand, when considering the entire set of proteins identified in the cellular secretome, rather than restricting the analysis to exclusively secreted proteins, a considerable number of cytoplasmic and nuclear proteins were detected as more abundant as the venom concentration increased and cell viability decreased. This shift likely resulted from the leakage of intracellular contents following phospholipid bilayer disruption or from cell death pathways directly or indirectly triggered by venom toxins. Using the ShinyGO 0.77 tool, we performed a functional enrichment analysis to identify biological processes associated with these proteomic alterations. The results revealed distinct modulatory patterns promoted by both of the venoms. In cells exposed to adult venom, we observed a predominant reduction in proteins associated with cytoskeletal organization, extracellular matrix (ECM), and supramolecular fiber organization, and RNA processing, whereas proteins related to metabolism and biomolecule modification were significantly increased. In contrast, cells treated with newborn venom exhibited a decrease in processes associated with blood vessel remodeling and immune signaling, alongside an increase in processes involving biomolecule metabolism, regulation of cell adhesion, and ECM organization, revealing a distinct functional signature compared to adult venom (). Cellular processes distinct from those of active protein secretion became more prevalent at higher venom concentrations. This newly emerging protein and peptide repertoire may have substantially influenced the homeostasis and signaling of neighboring viable cells, adding a layer of complexity beyond the direct action of toxins.

### 3.4 Semispecific Analysis Reveals Enhanced Proteolytic Processing Following Venom Exposure

To investigate proteolytic activity in the conditioned medium, secretome MS/MS data from cells exposed to adult and newborn *B. jararaca* venom were subjected to a semispecific database search using InfinDIA module of DIA-NN (). This approach aimed to identify cleavage patterns distinct from canonical tryptic peptides, which consistently contain arginine or lysine at the C-terminus. Consequently, the semispecific peptides detected in the secretome likely originate from the proteolytic activity of either the venom or the host cells. In cells incubated with adult venom, a clear trend toward an increased number of nontryptic peptides was observed as venom concentration increased (Figure 7A). In contrast, for newborn snake venom, only the 4 μg/mL venom condition showed a statistically significant increase compared to the control (Figure 7B). These differences in proteolytic activity between adult and newborn venoms have also been reported in substrate-specific assays,^22^ in which the proteolytic activity of adult venom was shown to be approximately 7-fold higher against collagen compared to newborn venom, while adult venom degraded bovine fibrinogen chains more rapidly. Likewise, Zelanis and colleagues^23^ reported higher proteolytic activity upon casein and fibrinogen by the adult venom.

**Figure 7:**
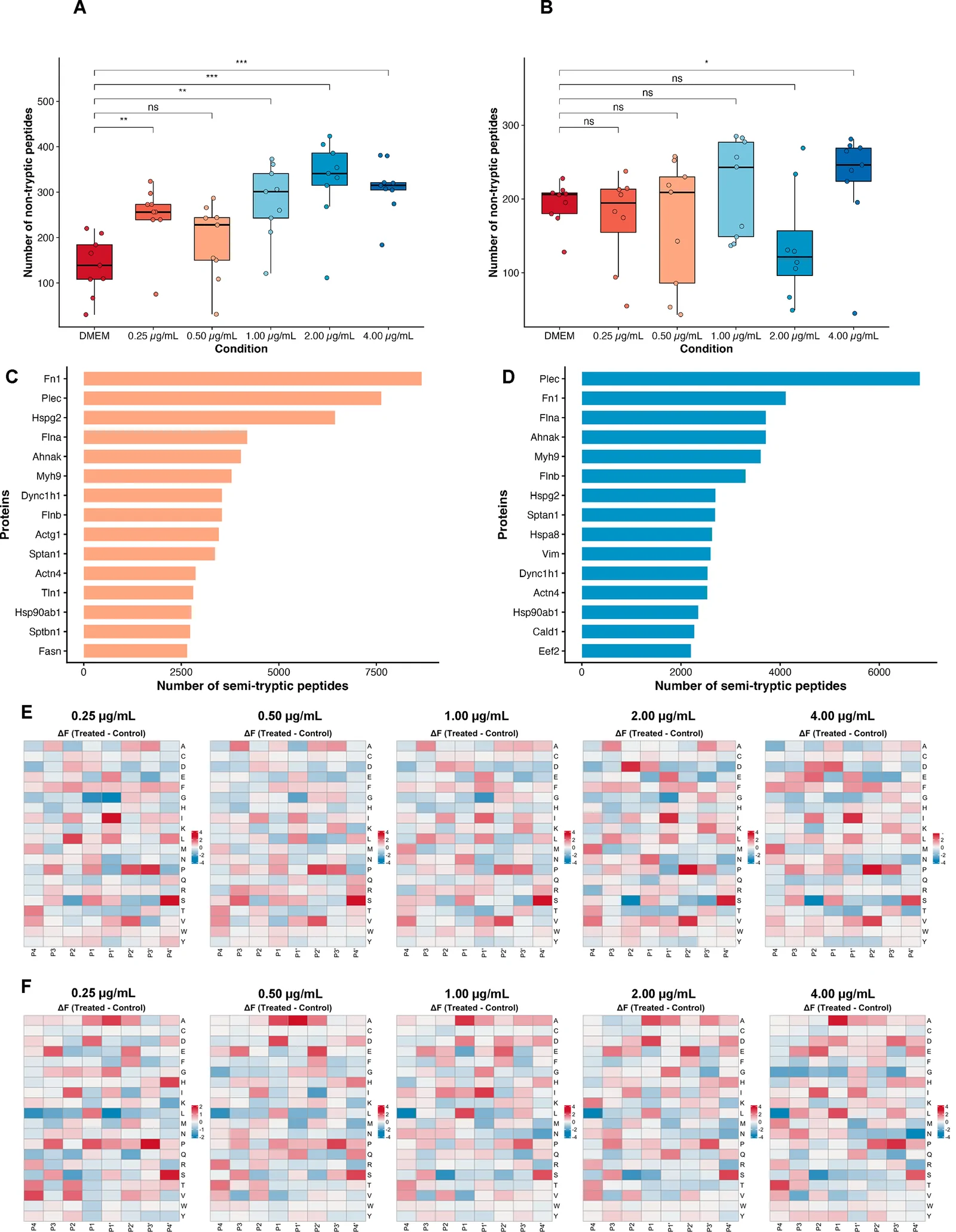
Semispecific peptide identification in LC-MS/MS secretome profiles. Number of nontryptic peptides identified in the cellular secretome of cells incubated with different concentrations of venom from adult (A) and newborn (B) *B. jararaca* specimens followed by Wilcoxon test. The top 15 proteins from which most semitryptic peptides were identified in secretome samples of cells treated with adult (C) and newborn (D) venom. Heat maps depicting the relative occurrence of each amino acid residue in proteolytic products ranging from P4–P4′ in peptides detected by the semispecific database search of secretome proteomics data of cells incubated with increased concentrations of *B. jararaca* adult (E) and newborn (F) venom for 2 h.

Analysis of the major cleavage targets revealed that the most affected proteins in the secretome were highly consistent between both venoms (Figure 7C,D). Notably, there was a clear predominance of peptides derived from plectin (PLEC), fibronectin-1 (FN1), and filamin A (FLNA). In some cases, differences in abundance ranking were observed; for example, the basement membrane-specific heparan sulfate proteoglycan core protein (HSPG2) was the third most represented degraded protein in the secretome of cells incubated with adult venom, whereas it ranked seventh in cells treated with newborn venom. Interestingly, under both conditions, HSPG2 showed reduced abundance within the cell proteome and increased abundance in the secretome, accompanied by enhanced proteolytic degradation, suggesting increased secretion followed by extracellular degradation, similar to what was observed for FN1. Most semitryptic peptides were derived from cytoskeletal, membrane-associated, and extracellular matrix proteins, as well as proteins related to translational processes. The generation of peptides derived from membrane-associated and extracellular matrix proteins was expected, as previously reported by Asega and colleagues^45,63^ since both venoms are rich in proteases displaying a broad substrate repertoire.

Nevertheless, analysis of the differential amino acid frequency at cleavage sites relative to control samples (Δfrequency: treated – control) (Figure 7E,F) as well as analysis of the frequency of each amino acid () revealed atypical proteolytic profiles. In cells exposed to lower concentrations of adult venom (0.25–1.0 μg/mL), the most frequent amino acids at the P1 and P1’ positions, corresponding to the residues flanking the cleavage site at the N- and C-terminal sides, were, respectively, serine (Ser) and isoleucine (Ile). This pattern strongly suggests the activity of cathepsin-like proteases (including cathepsins E, V, L, S, K, D, and B), calpain-1 and -2, and matrix metalloproteases (MMPs). Because these enzymes are absent from the venom proteome itself, they are likely cellular in origin. Cells incubated with newborn venom in the same concentration range displayed a shift in cleavage preference, predominantly toward aspartic acid (Asp) and alanine (Ala), again suggesting the activity of cysteine proteases such as cathepsins and legumain, in addition to caspases and matrix metalloproteases.

At higher venom concentrations (2.0 and 4.0 μg/mL), adult venom induced a slight shift in cleavage specificity toward aspartic acid (Asp) at the P1 position and isoleucine (Ile) at the P1’ position, consistent with the activity of cathepsins, legumain, caspases, metalloproteases, and additional proteases such as meprin alpha. A comparable trend was observed for newborn venom, which was mainly characterized by an enrichment of aspartic acid and leucine (Leu) at the P1 position. Consistently, profiles of *B. jararaca* venom-induced proteolysis in murine models have shown the *in vivo* activation of endogenous proteases, especially cathepsins.^64,30^

## 4 Conclusions

The comparative analysis of the effects of *B. jararaca* adult and newborn venoms on SV40 MES13 kidney cells suggests that ontogenetic variations profoundly dictate the mechanisms and severity of envenomation-induced nephrotoxicity. When comparing age profiles, adult venom exhibited a substantially higher cytotoxic potency than newborn venom, driving a reduction in cell viability within a short time frame. This difference likely reflects an ontogenetic shift in venom composition targeting age-specific prey, rather than an evolved resistance in nonprey mammalian tissues. Consequently, murine kidney cells exhibit a lower relative sensitivity to newborn venom, consistent with a venom system optimized for juvenile prey rather than mammalian hosts.

At the molecular level, adult venom exerted a much more aggressive proteolytic demand on cell culture. It targeted components of cell architecture by directly degrading structural proteins, driving a phenotypic transition toward a pro-fibrotic state. Conversely, newborn venom elicited a significantly more modest, gradual, and delayed cellular stress response. This is likely because in bothropic envenomation, the proteases in newborn venom preferentially target components of the coagulation cascade rather than extracellular matrix proteins, as is the case for adult venom.

For the adult venom, when evaluating the experimental compartments, the cellular content proteome revealed a distinct silent phase of damage at lower concentrations, where cells remained viable, but their internal survival machinery was already compromised and primed for death. As concentrations increased, the cellular proteome demonstrated a clear collapse of cytoskeletal and adhesion structures. In contrast, the newborn venom had a negligible impact on the cellular proteome profile at low-to-mid concentrations, only triggering a significant proteomic shift at the highest concentration (4.0 μg/mL). Furthermore, instead of a collapse, cells exposed to newborn venom showed an increase in the abundance of these structural and adhesion proteins in the secretome as a stress response.

In contrast, the secretome profile provided an even more pronounced discrimination of venom-induced effects, capturing the dynamic nature of the extracellular environment with distinct age-related signatures. Under the influence of adult venom, the secretome was heavily shaped by extensive extracellular matrix degradation, membrane shedding, and a substantial influx of intracellular contents, resulting from cell lysis. Conversely, newborn venom elicited a more gradual, dose-dependent change in the secretome, predominantly characterized by an increased abundance of naturally secreted proteins, reflecting an active cellular stress response.

Furthermore, semispecific peptide searches within the secretome revealed that while adult venom caused broad proteolytic processing of major structural targets, both venoms triggered a proteolytic cascade by activating distinct classes of endogenous host proteases, demonstrating that the cells were eventually forced to participate in their own structural disassembling.

Both cell content and the secretome showed a dependence on increased venom concentrations. At low doses of adult venom, the cells entered a quiet phase where their internal survival machinery was compromised, while they remained viable, whereas low doses of newborn venom have a negligible impact. As the concentrations increased, the system was rapidly pushed beyond its limits. This dose-dependent progression drove a divergent profile between the compartments, where the depletion of structural proteins within the cell content was mirrored by an accumulation of degradation products in the secretome.

Our findings serve as a proof-of-concept demonstrating how distinct functional venom phenotypes (adult vs newborn) differentially modulate renal cell responses and secretome profiles. Because the adult venom pool encompasses specimens from both southeastern and southern regions of Brazil, potential geographic or population-level variations cannot be entirely distinguished from pure ontogenetic shifts in this data set. Future studies utilizing lineage-matched and geographically controlled venom pairs (e.g., SE-newborn vs SE-adult) will be essential to isolate age-dependent nephrotoxic mechanisms from population-specific proteomic signatures.

This study underscored the great value of multiomic, multicompartment *in vitro* modeling to decipher how complex snake venom variations translate into tissue-specific pathologies, paving the way for more targeted therapeutic strategies.

## Supplementary Material





## Data Availability

All mass spectrometry proteomics data have been deposited to the ProteomeXchange Consortium (http://proteomecentral.proteomexchange.org), via the PRIDE partner repository (https://www.ebi.ac.uk/pride/) with project accession PXD079314.

## Notes

Supplementary Figure S1. Schematic illustration of the experimental design used for sample preparation for the proteomic analyses. Supplementary Figure S2. Chromatographic elution profiles of precursor ions and distribution of missing protein values in samples obtained from the cellular content of cells incubated with different concentrations of adult (A) or newborn (B) *B. jararaca* venom for 2 h. Supplementary Figure S3. Number of proteins identified in each sample from the cellular content of cells incubated with adult (A) or newborn (B) *B. jararaca* venom for 2 h. Supplementary Figure S4. Distribution of raw intensity across samples from the cellular content of cells incubated with adult (A) or newborn (B) *B. jararaca* venom for 2 h. Supplementary Figure S5. Distribution of the coefficients of variation (%) for proteins under each experimental condition in samples obtained from the cellular content of cells incubated with adult (A) or newborn (B) *B. jararaca* venom for 2 h. Supplementary Figure S6. Chromatographic elution profiles of precursor ions and distribution of missing protein values in samples obtained from the cellular secretome of cells incubated with different concentrations of adult (A) or newborn (B) *B. jararaca* venom for 2 h. Supplementary Figure S7. Number of proteins identified in each sample from the cellular secretome of cells incubated with adult (A) or newborn (B) *B. jararaca* venom for 2 h. Supplementary Figure S8. Distribution of raw intensity across samples from the cellular secretome of cells incubated with adult (A) or newborn (B) *B. jararaca* venom for 2 h. Supplementary Figure S9. Distribution of the coefficients of variation (%) for proteins under each experimental condition in samples obtained from the cellular secretome of cells incubated with adult (A) or newborn (B) *B. jararaca* venom for 2 h. Supplementary Figure S10. Enrichment of biological processes for proteins with altered abundance in the secretome of SV40 MES13 cells exposed to adult *B. jararaca* venom for 2 h. Supplementary Figure S11. Enrichment of biological processes for proteins with altered abundance in the secretome of SV40 MES13 cells exposed to newborn *B. jararaca* venom for 2 h. Supplementary Figure S12. Heat maps depicting the relative occurrence of each amino acid residue within proteolytic products (peptides) spanning positions P4–P4′ in the cellular secretome from the cells exposed to adult (A) or newborn (B) *B. jararaca* venom for 2 h. (PDF)
Supplementary Table S1. Summary of acquired and excluded LC-MS/MS proteomic samples from SV40 MES13 cells after 2 h incubation with adult or newborn *B. jararaca* venom. Supplementary Table S2. Differentially abundant proteins of the cellular content of SV40 MES13 cells incubated with adult *B. jararaca* venom for 2 h. Supplementary Table S3. Differentially abundant proteins of the cellular content of SV40 MES13 cells incubated with newborn *B. jararaca* venom for 2 h. Supplementary Table S4. Differentially abundant proteins of the secretome of SV40 MES13 cells incubated with adult *B. jararaca* venom for 2 h. Supplementary Table S5. Differentially abundant proteins of the secretome of SV40 MES13 cells incubated with newborn *B. jararaca* venom for 2 h. Supplementary Table S6. Semispecific search of LC-MS/MS spectra of secretome of SV40 MES13 cells incubated with adult *B. jararaca* venom for 2 h. Supplementary Table S7. Semispecific search of LC-MS/MS spectra of secretome of SV40 MES13 cells incubated with newborn *B. jararaca* venom for 2 h. (ZIP)
