# Supplementary material for: Ontogenetic Shift in a Snake Venom Proteome Drives Distinct Proteomic Responses in Mouse Renal Mesangial Cells
Source: J Proteome Res. 2026 Oct 15;25(11):6094–114. doi: 10.1021/acs.jproteome.6c00503 (PMC13750073; doi:10.1021/acs.jproteome.6c00503)
Supplement: Supplementary file 1 [file pr6c00503_si_002.pdf]

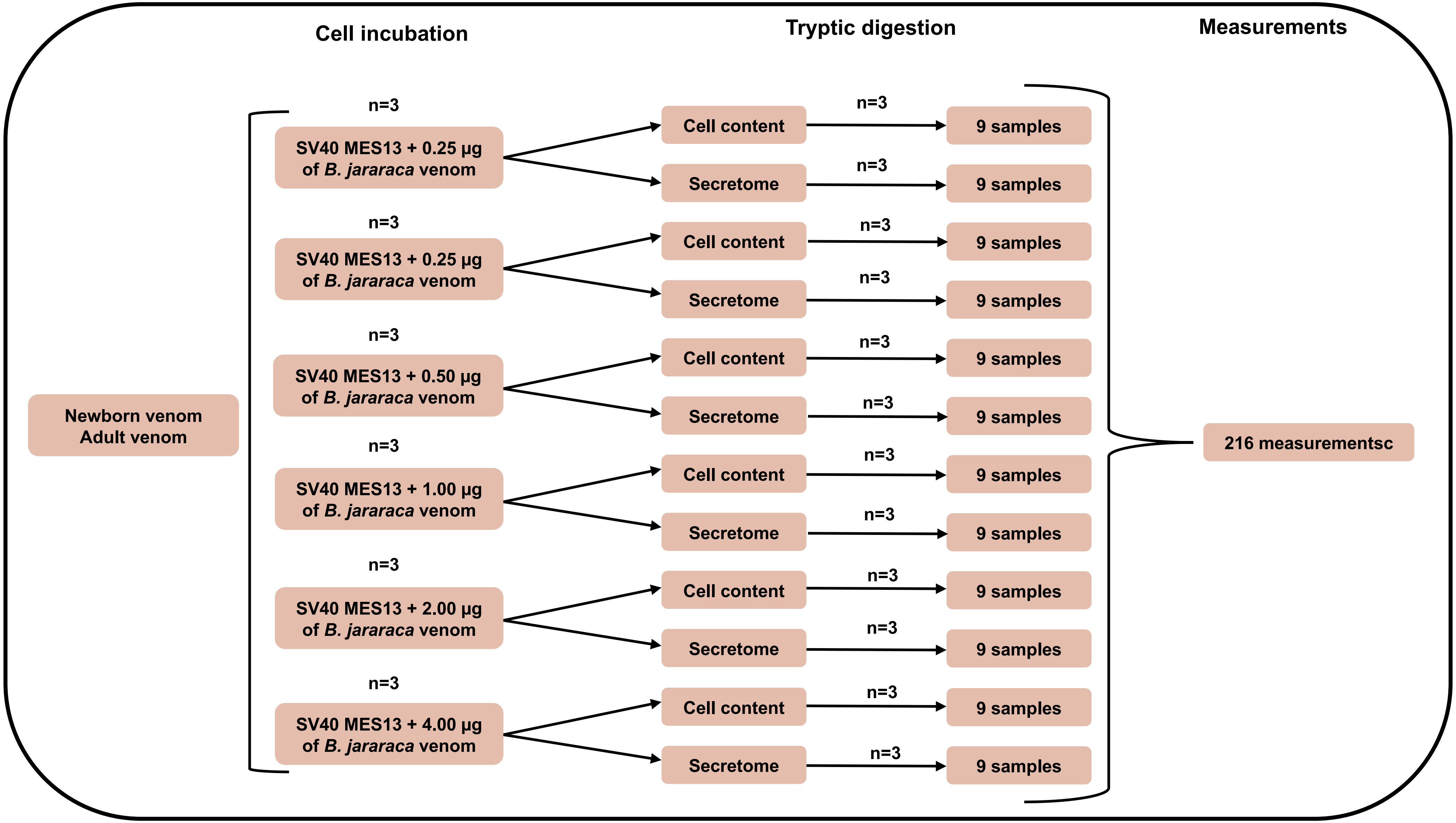

Supplementary Figure S1. Schematic illustration of the experimental design used for sample preparation for the proteomic analyses. Incubation experiments were performed in triplicate for each concentration of adult and newborn *Bothrops jararaca* venom and were designated for proteomic analyses of both the cellular content and the secretome. In addition, protein digestions were also carried out in triplicate, and each digest was individually analyzed by LC–MS/MS.

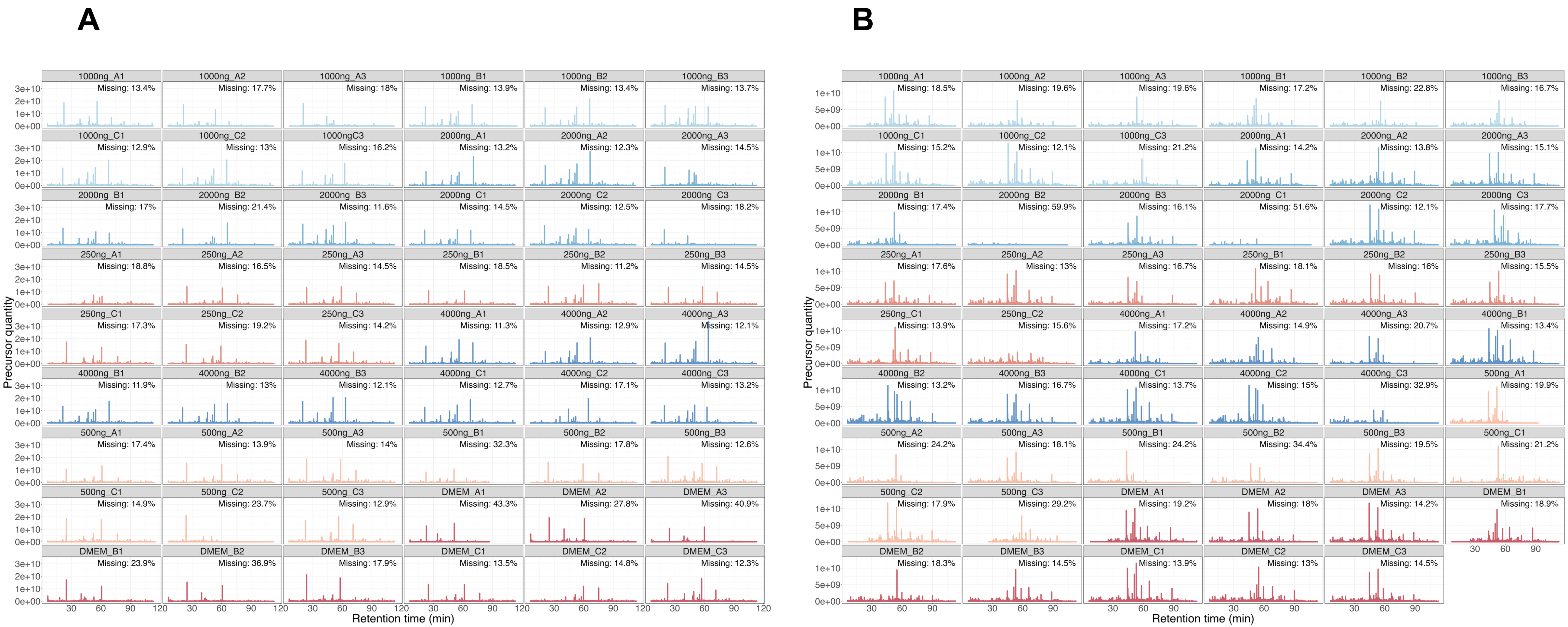

Supplementary Figure S2. Chromatographic elution of precursor ions and distribution of protein missing values in the samples. Samples were obtained from the cellular content of cells incubated with different concentrations of adult (A) or newborn (B) *Bothrops jararaca* venom for 2 h. Samples correspond to cell cultures incubated for 2 h with different concentrations of *Bothrops jararaca* venom: 250ng_X (0.25 μg venom), 500ng_X (0.5 μg venom), 1000ng_X (1.0 μg venom), 2000ng_X (2.0 μg venom), and 4000ng_X (4.0 μg venom) and the control DMEM (0.15 M NaCl).

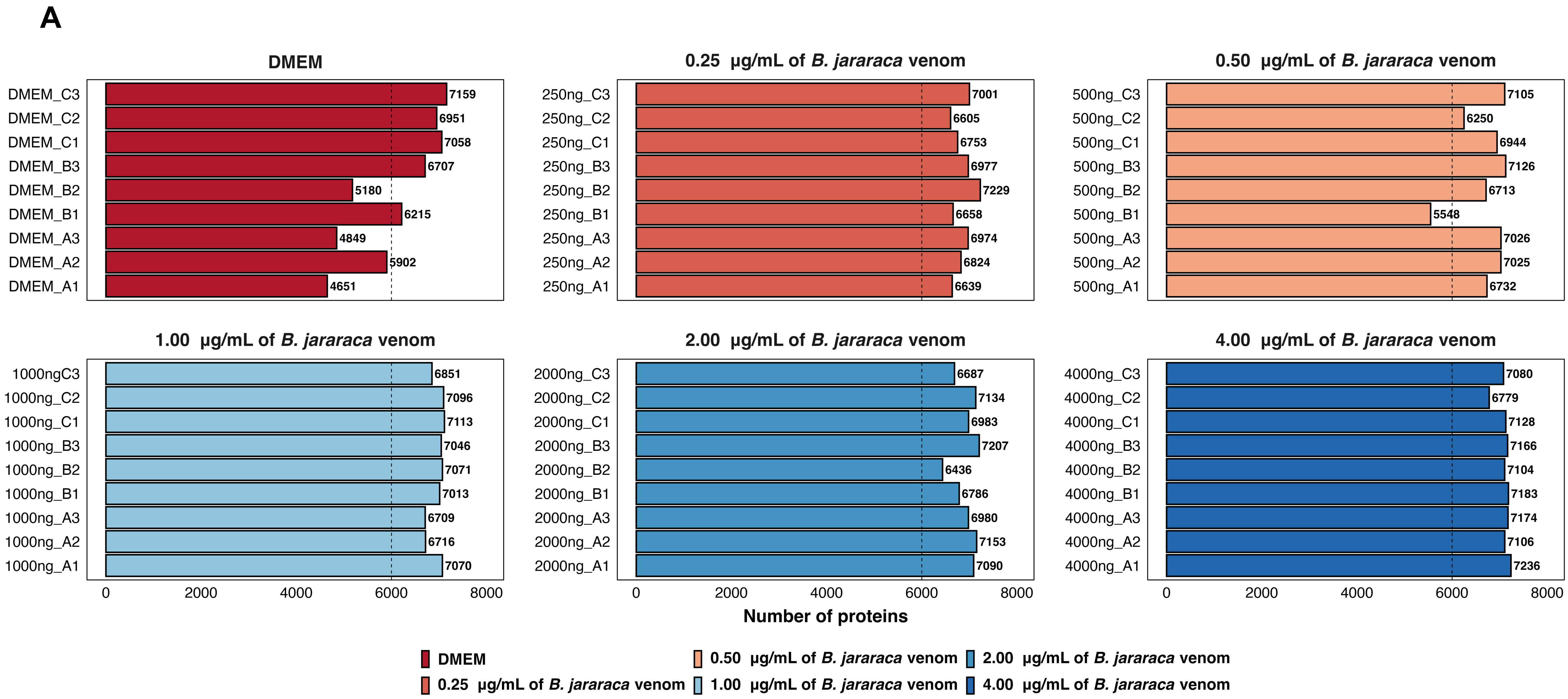

Supplementary Figure S3. Number of proteins identified in each sample. Bar graph showing the number of unique proteins identified across all samples and conditions from the cellular content of cells incubated with adult (A) or newborn (B) *Bothrops jararaca* venom for 2 h.

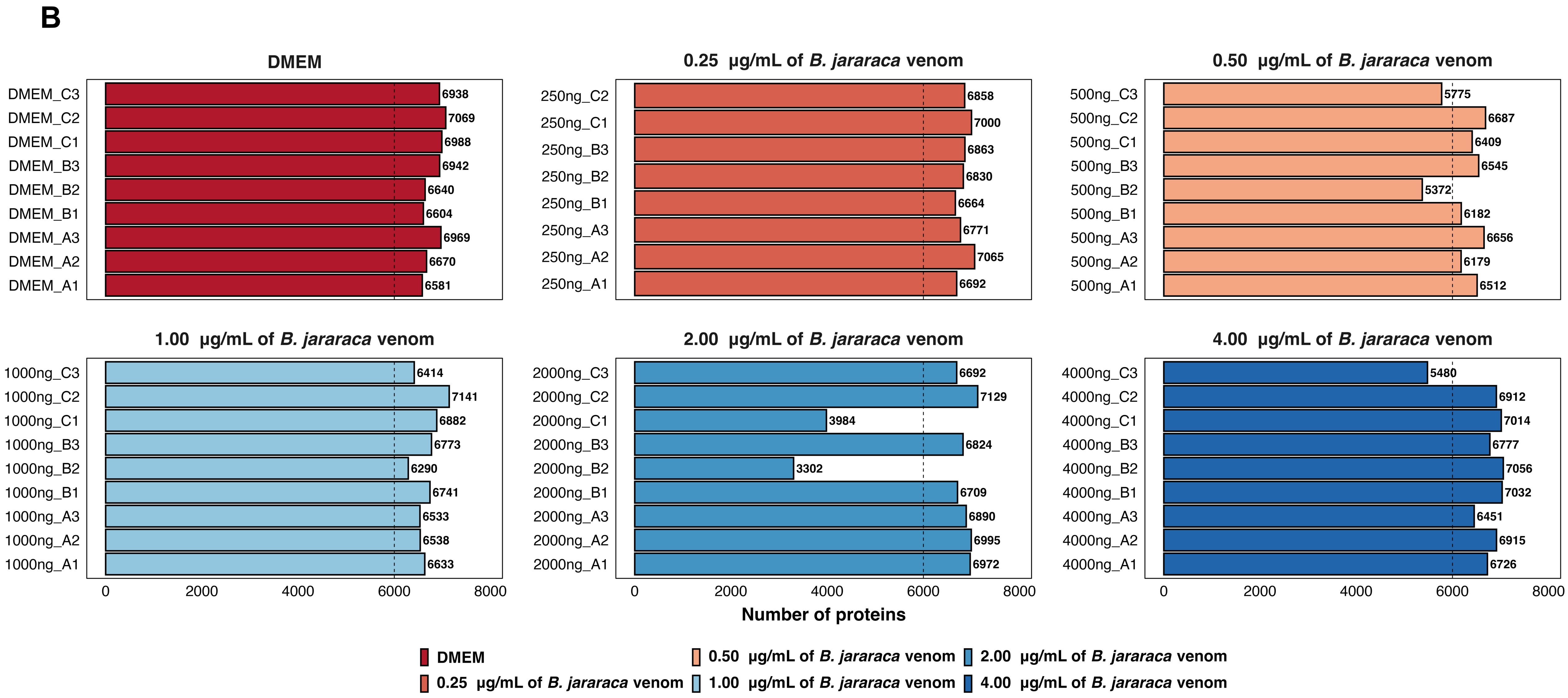

Supplementary Figure S3. Number of proteins identified in each sample. Bar graph showing the number of unique proteins identified across all samples and conditions from the cellular content of cells incubated with adult (A) or newborn (B) *Bothrops jararaca* venom for 2 h.

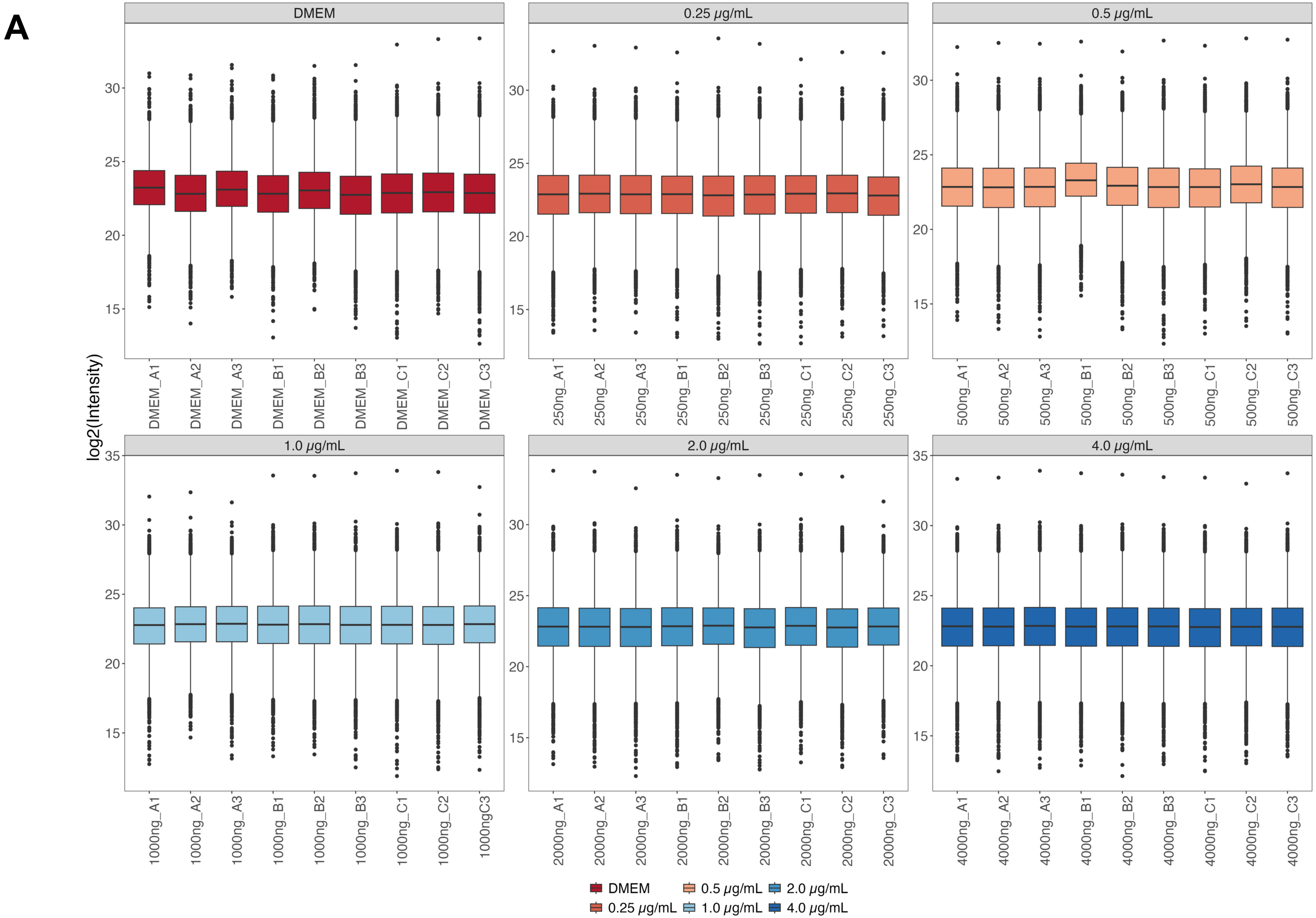

Supplementary Figure S4. Distribution of raw intensity across samples. Box plot illustrating the distribution of log2-transformed intensity values among the different samples from the cellular content of cells incubated with adult (A) or newborn (B) *Bothrops jararaca* venom for 2 h.

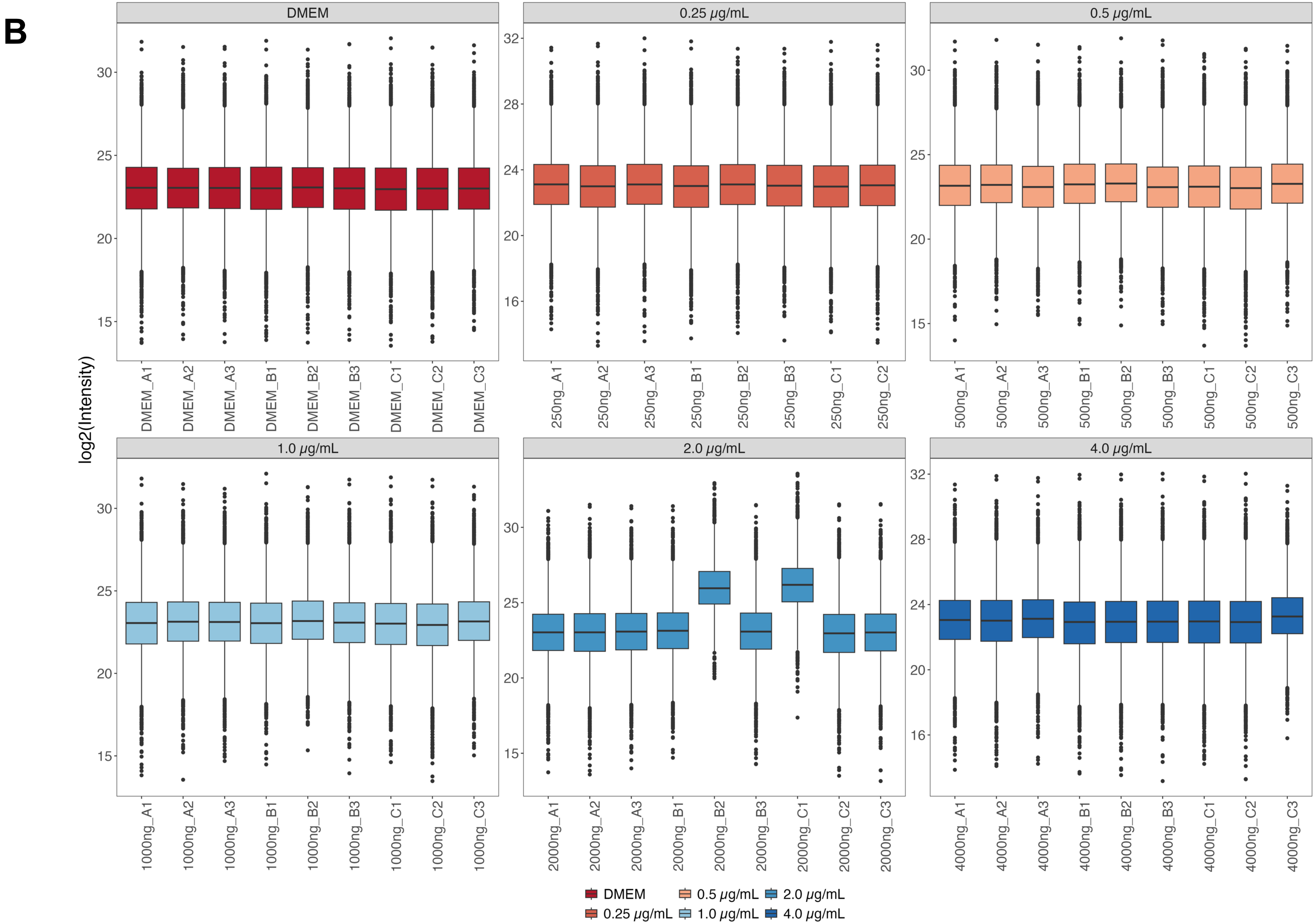

Supplementary Figure S4. Distribution of raw intensity across samples. Box plot illustrating the distribution of log2-transformed intensity values among the different samples from the cellular content of cells incubated with adult (A) or newborn (B) *Bothrops jararaca* venom for 2 h.

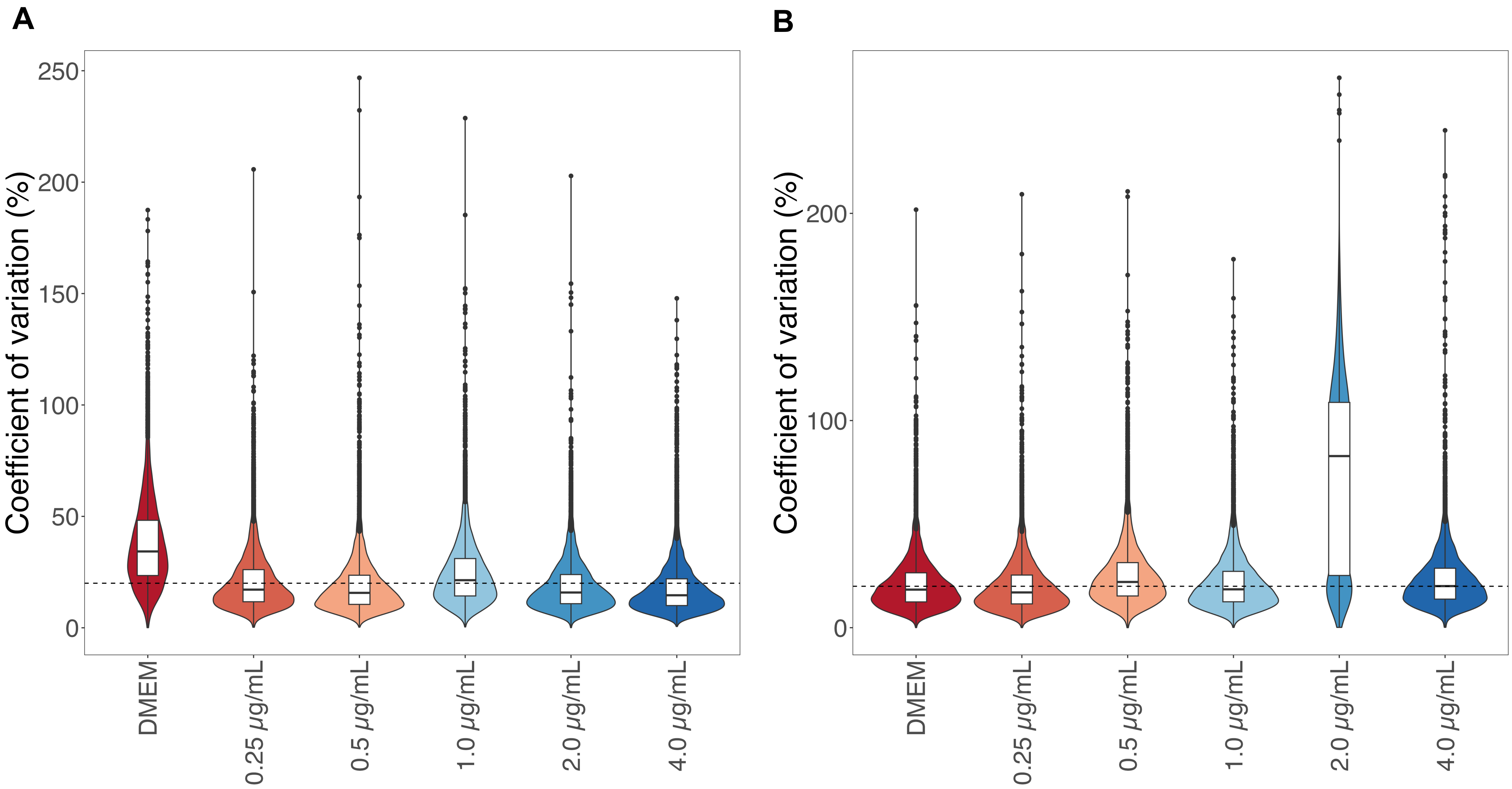

Supplementary Figure S5. Distribution of coefficients of variation (%) for proteins in each condition. Samples were obtained from the cellular content of cells incubated with adult (A) or newborn (B) *Bothrops jararaca* venom for 2 h. The dashed line indicates 20%, representing an estimate of good precision in proteomic analyses.

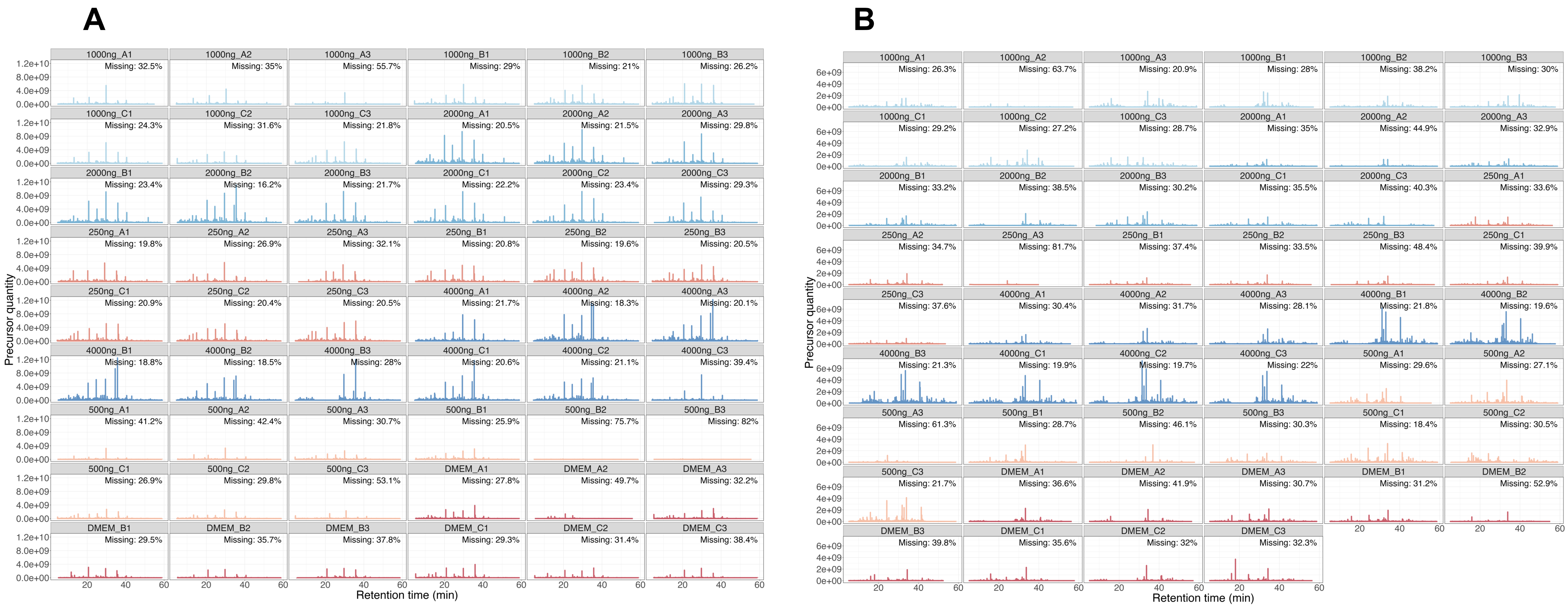

Supplementary Figure S6. Chromatographic elution of precursor ions and distribution of protein missing values in the samples. Samples were obtained from the secretome of cells incubated with different concentrations of adult (A) or newborn (B) *Bothrops jararaca* venom for 2 h. Samples correspond to cell cultures incubated for 2 h with different concentrations of *Bothrops jararaca* venom: 250ng_X (0.25 µg venom), 500ng_X (0.5 µg venom), 1000ng_X (1.0 µg venom), 2000ng_X (2.0 µg venom), and 4000ng_X (4.0 µg venom) and the control DMEM (0.15 M NaCl).

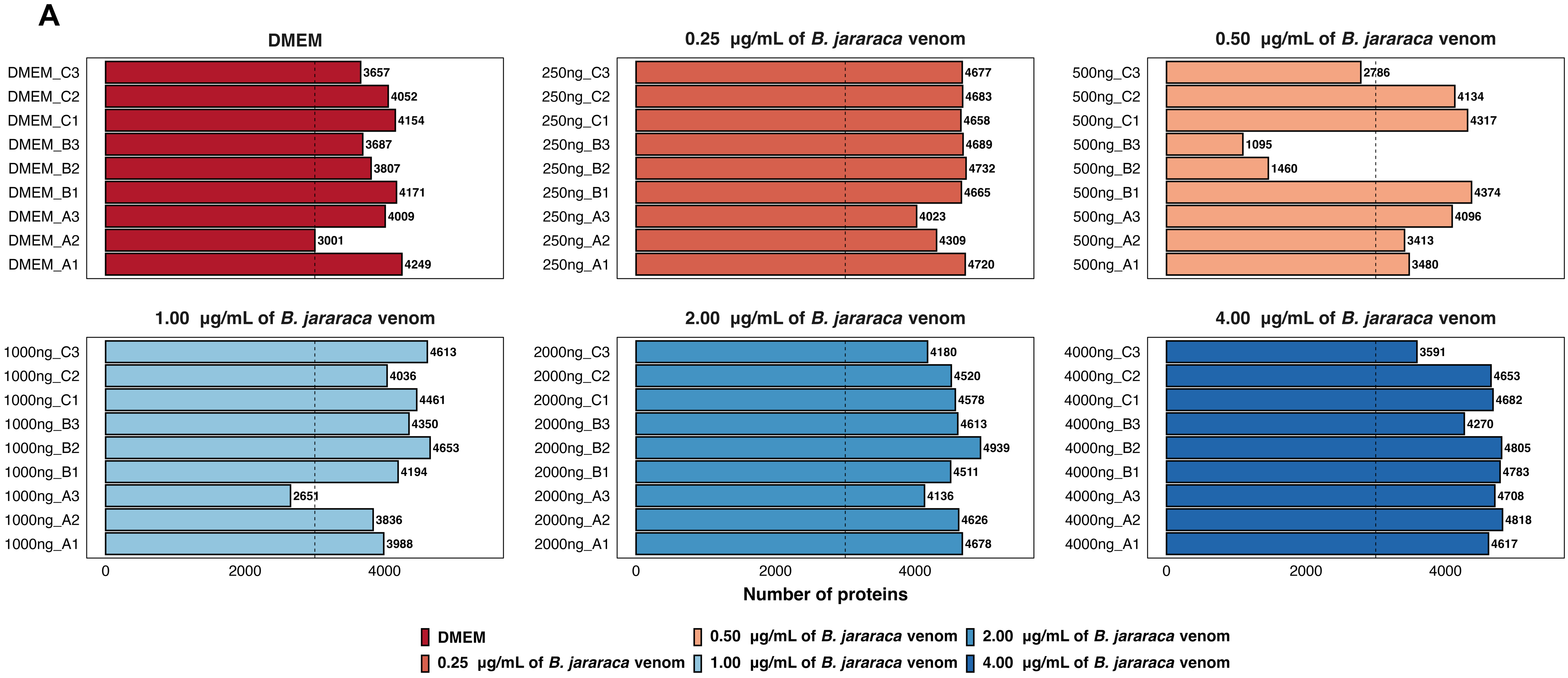

Supplementary Figure S7. Number of proteins identified in each sample. Bar graph showing the number of unique proteins identified across all samples and conditions from the secretome of cells incubated with adult (A) or newborn (B) *Bothrops jararaca* venom for 2 h.

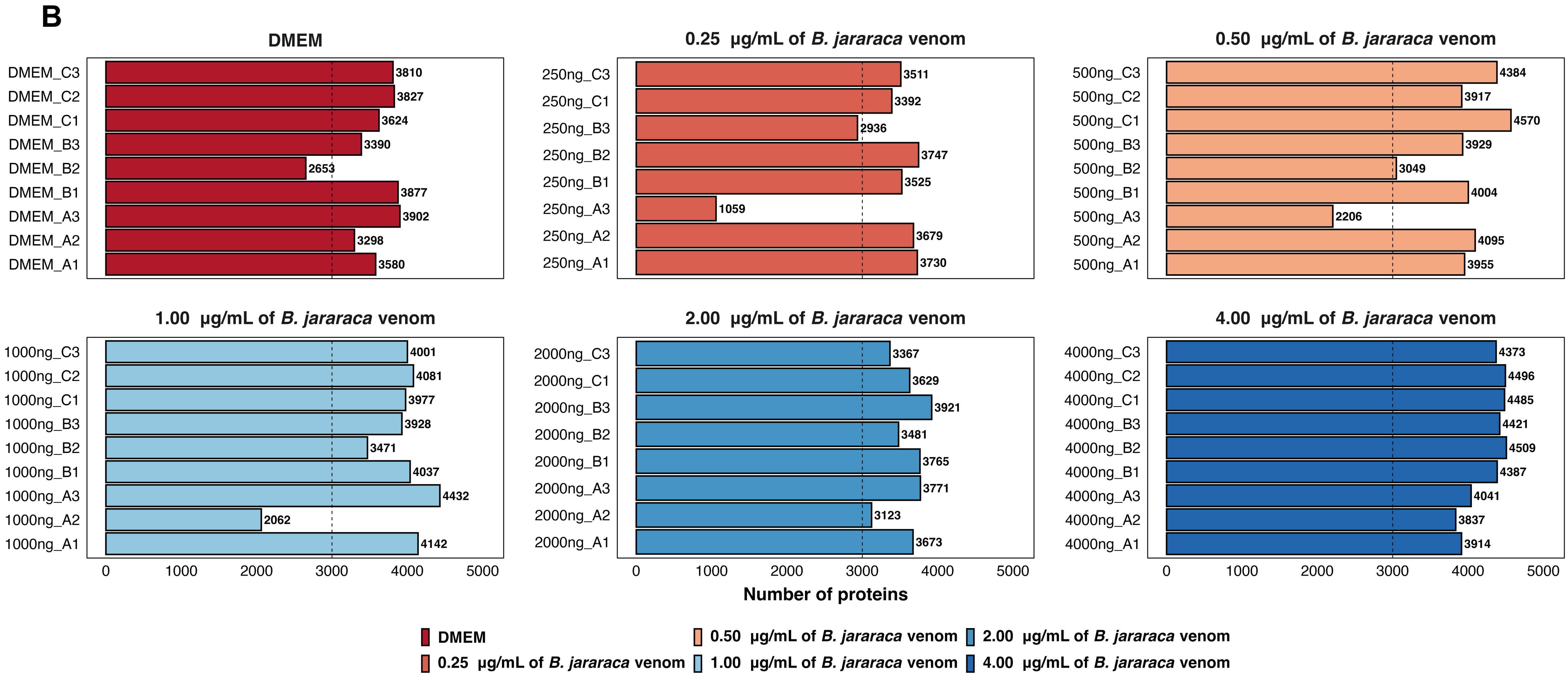

Supplementary Figure S7. Number of proteins identified in each sample. Bar graph showing the number of unique proteins identified across all samples and conditions from the secretome of cells incubated with adult (A) or newborn (B) *Bothrops jararaca* venom for 2 h.

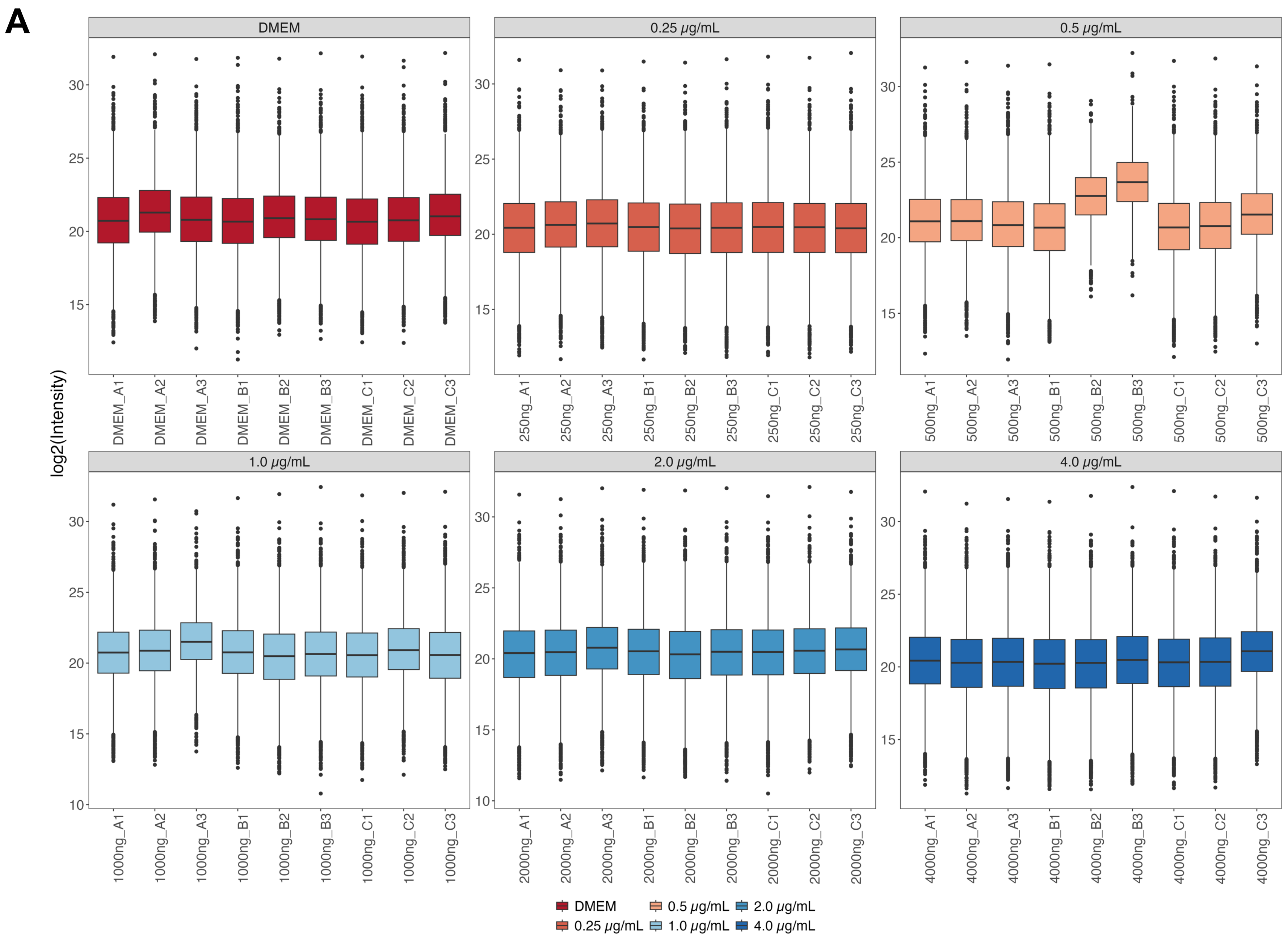

Supplementary Figure S8. Distribution of raw intensity across samples. Box plot illustrating the distribution of log2-transformed intensity values among the different samples from the secretome of cells incubated with adult (A) or newborn (B) *Bothrops jararaca* venom for 2 h.

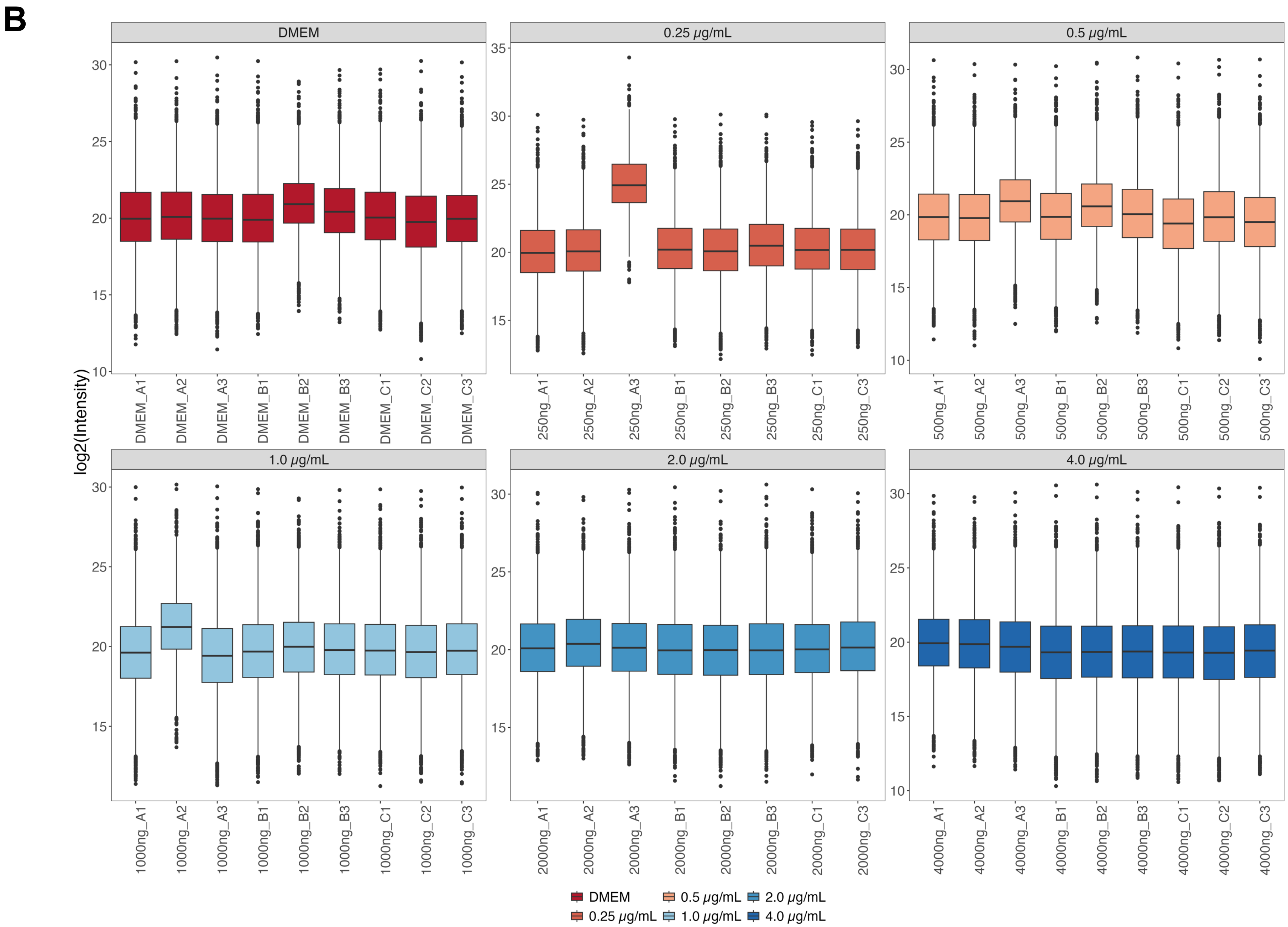

Supplementary Figure S8. Distribution of raw intensity across samples. Box plot illustrating the distribution of log2-transformed intensity values among the different samples from the secretome of cells incubated with adult (A) or newborn (B) *Bothrops jararaca* venom for 2 h.

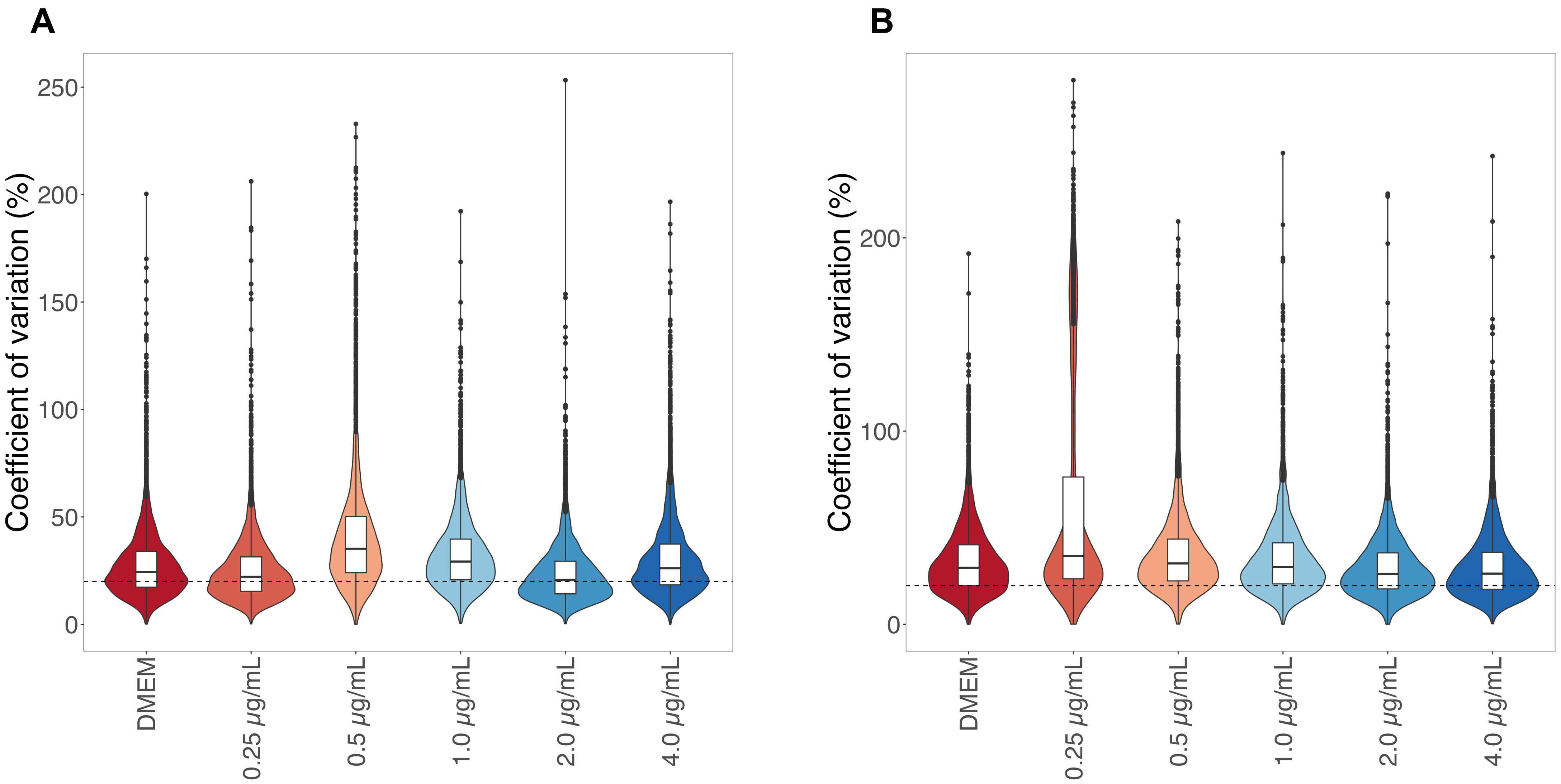

Supplementary Figure S9. Distribution of coefficients of variation (%) for proteins in each condition. Samples were obtained from the secretome of cells incubated with adult (A) or newborn (B) *Bothrops jararaca* venom for 2 h. The dashed line indicates 20%, representing an estimate of good precision in proteomic analyses.

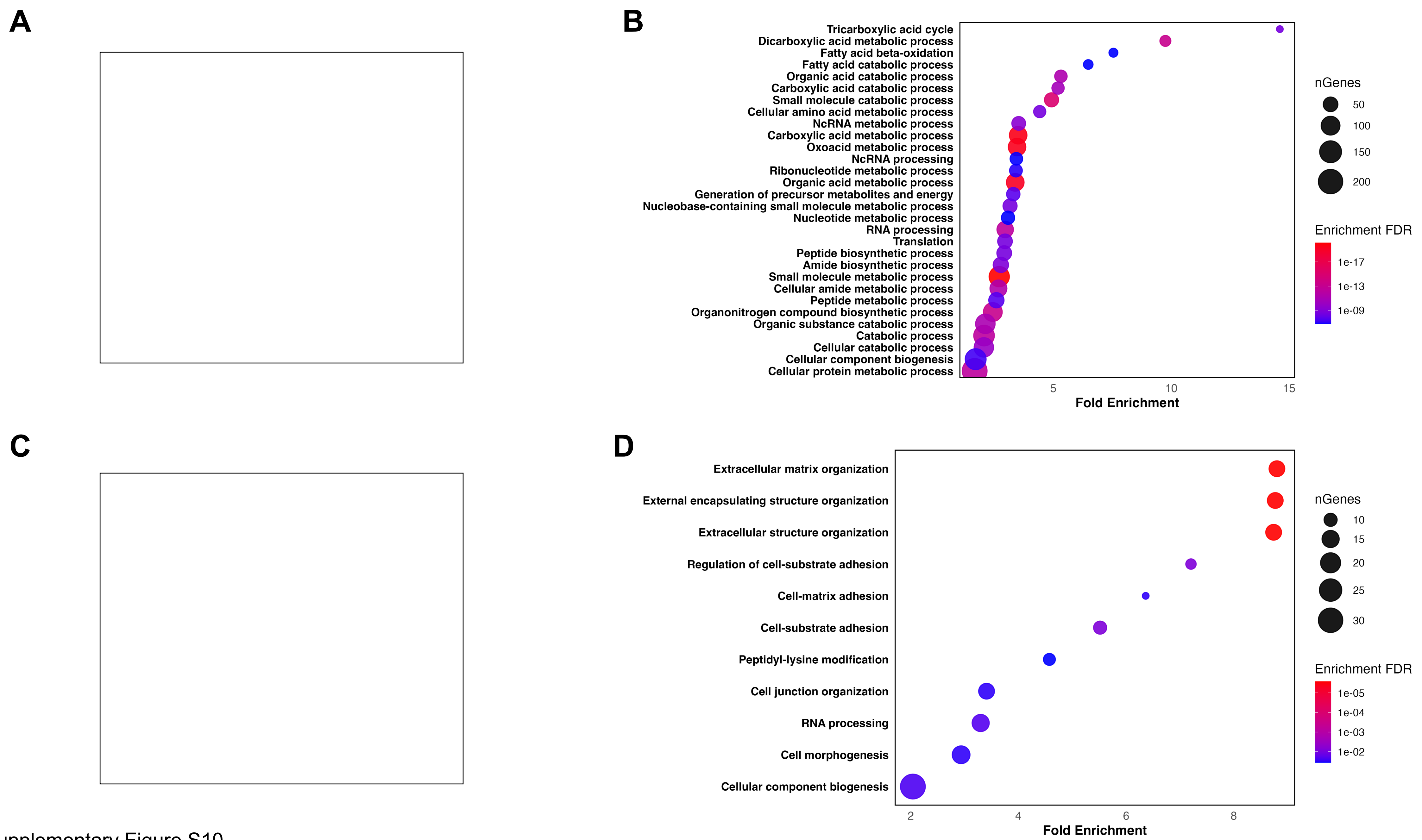

Supplementary Figure S10

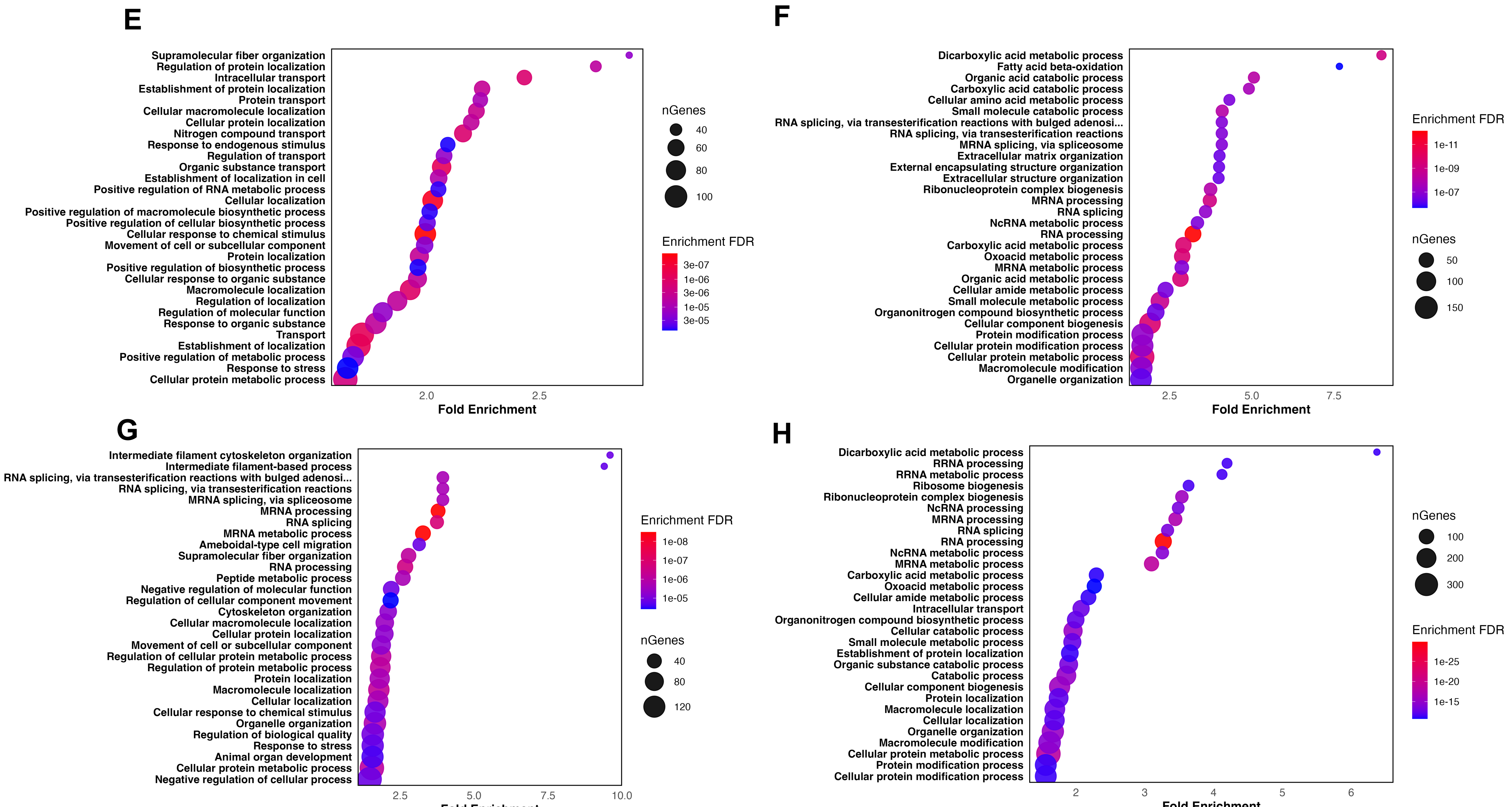

Supplementary Figure S10

I

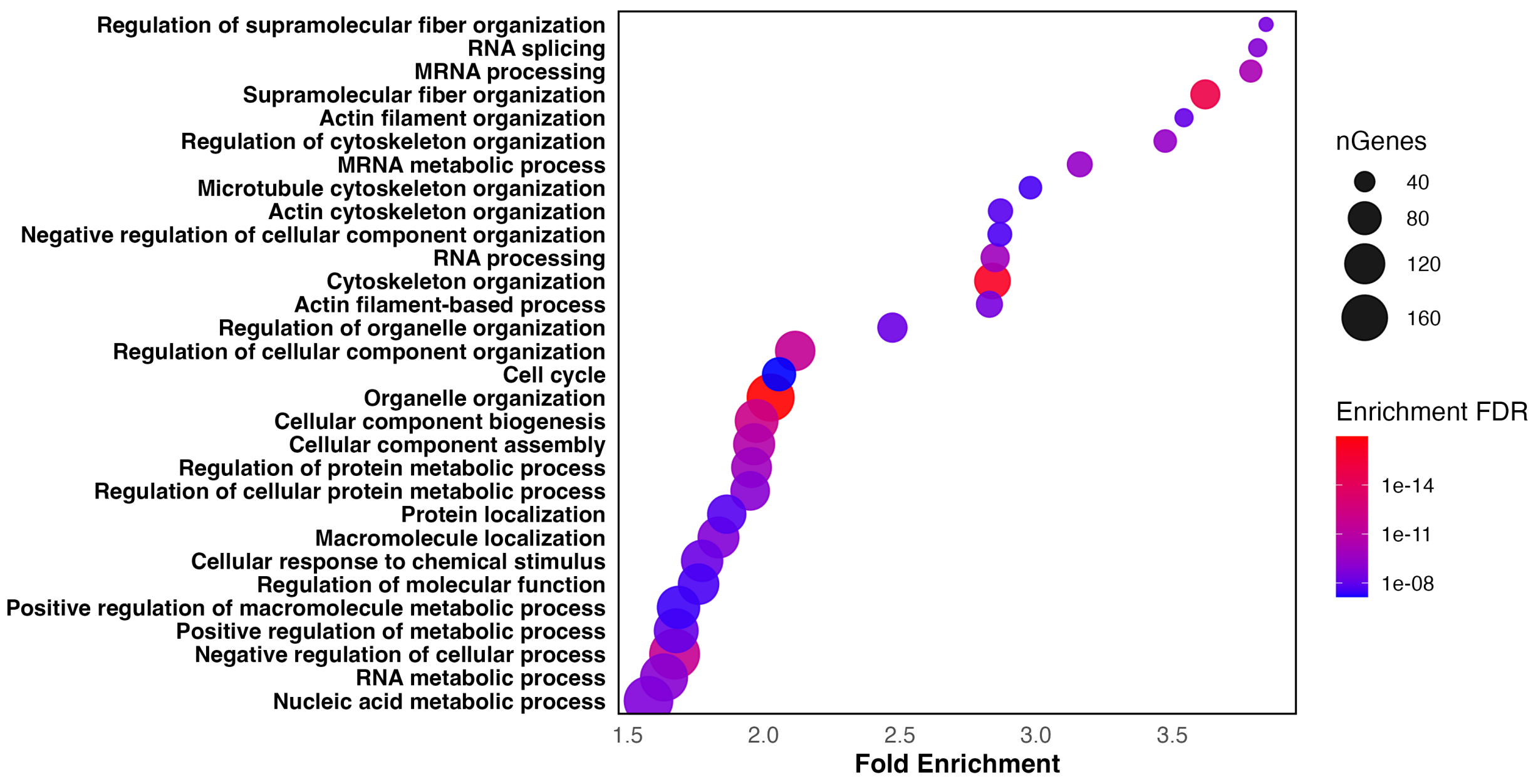

J

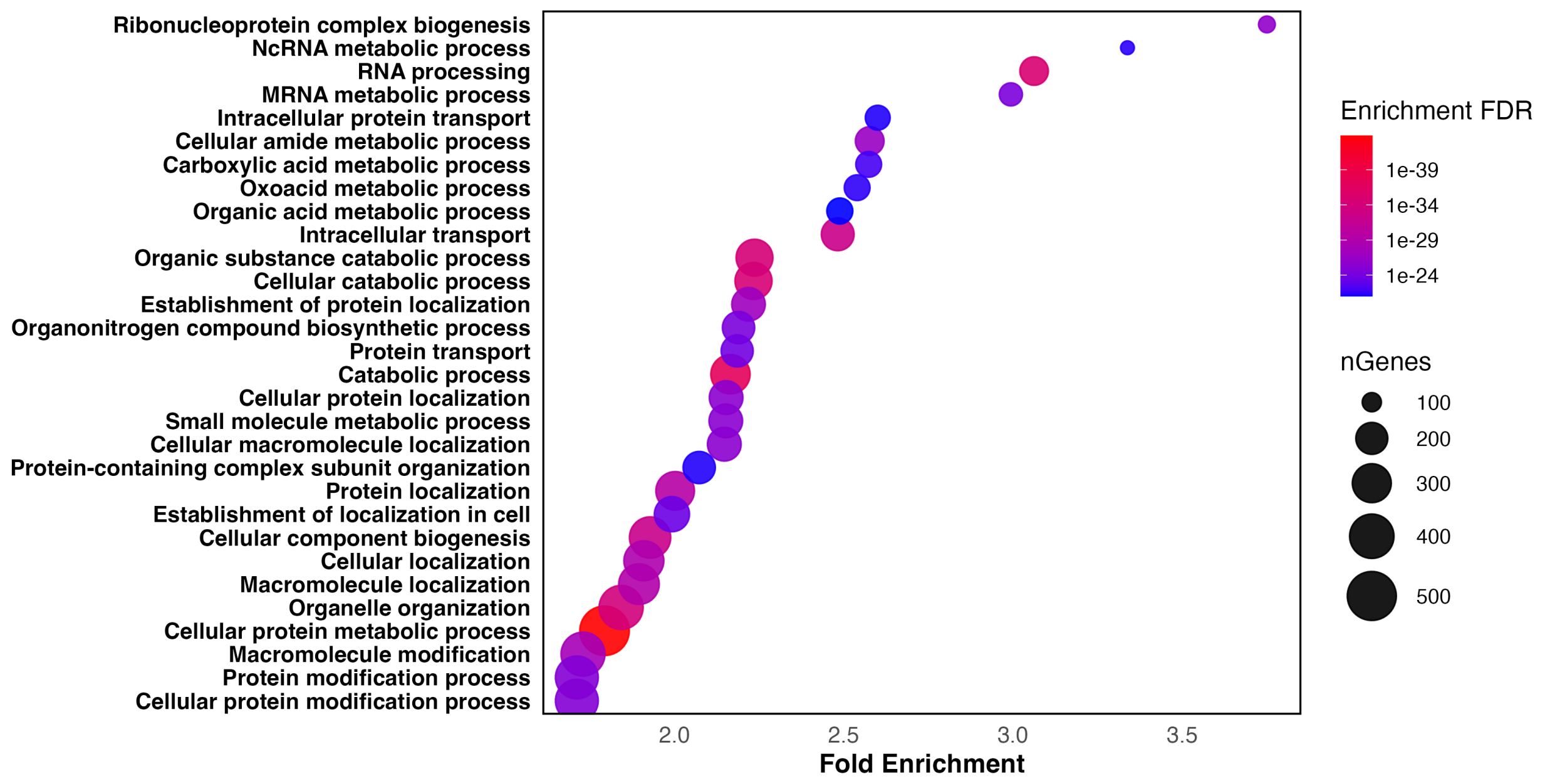

Supplementary Figure S10. Enrichment of biological processes for proteins with altered abundance in the secretome of SV40 MES13 cells exposed to adult *Bothrops jararaca* venom for 2 h. Biological enrichment showed for proteins with reduced (A) or increased (B) abundance following exposure to 0.25 µg/mL of venom; reduced (C) or increased (D) abundance at 0.50 µg/mL; reduced (E) or increased (F) abundance at 1.0 µg/mL; reduced (G) or increased (H) abundance at 2.0 µg/mL; and reduced (I) or increased (J) abundance at 4.0 µg/mL of adult *B. jararaca* venom.

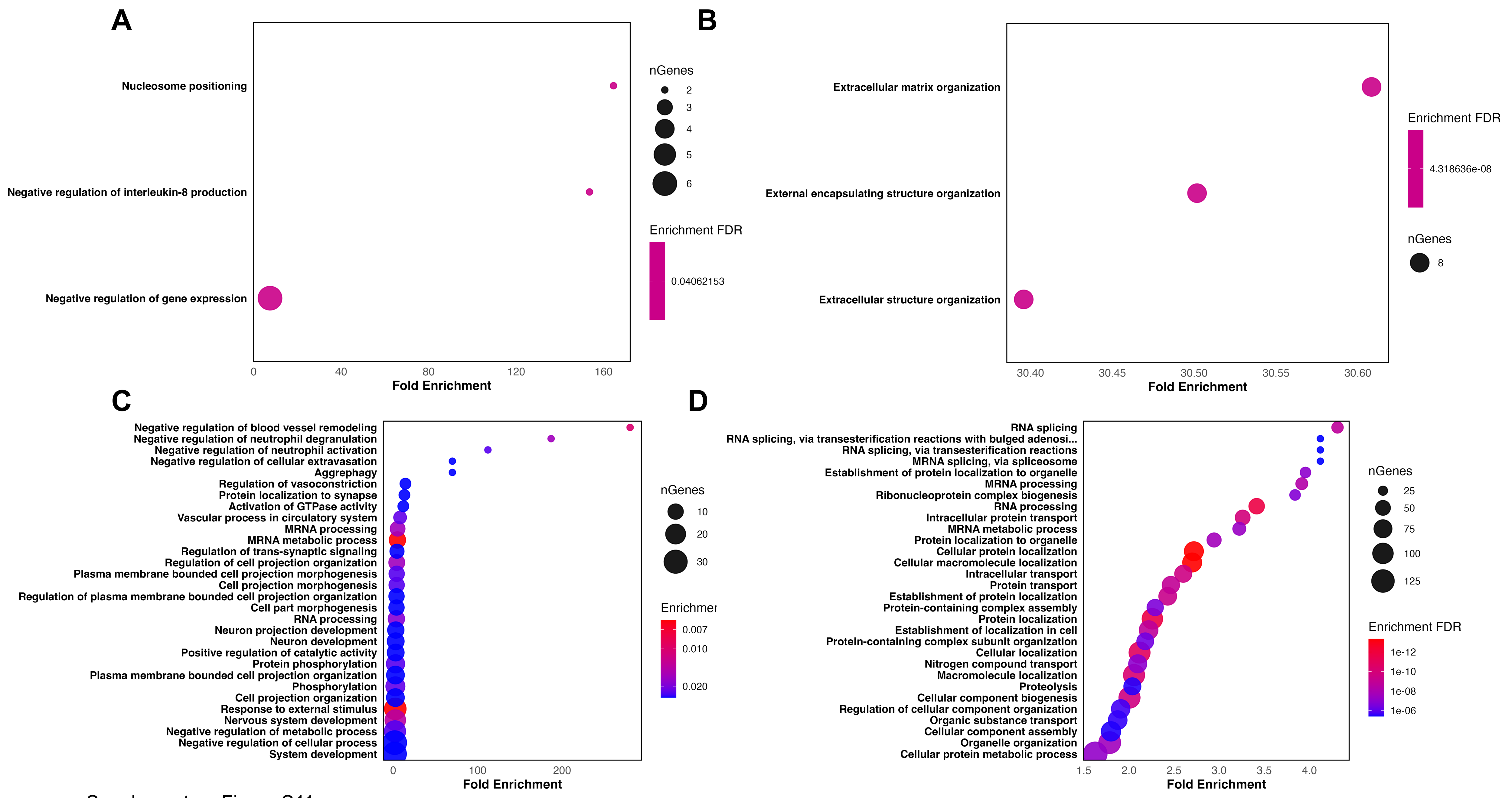

Supplementary Figure S11

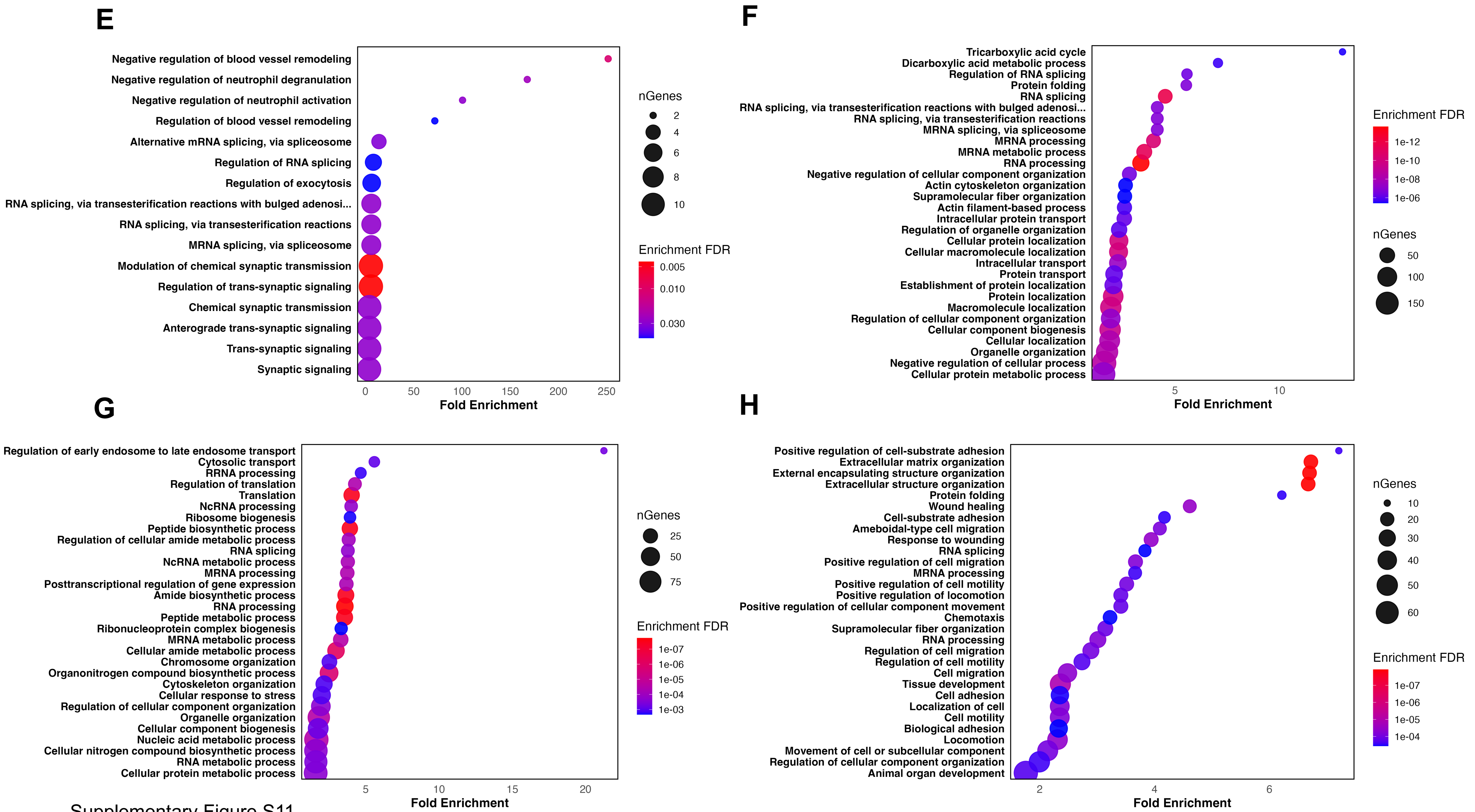

Supplementary Figure S11

I

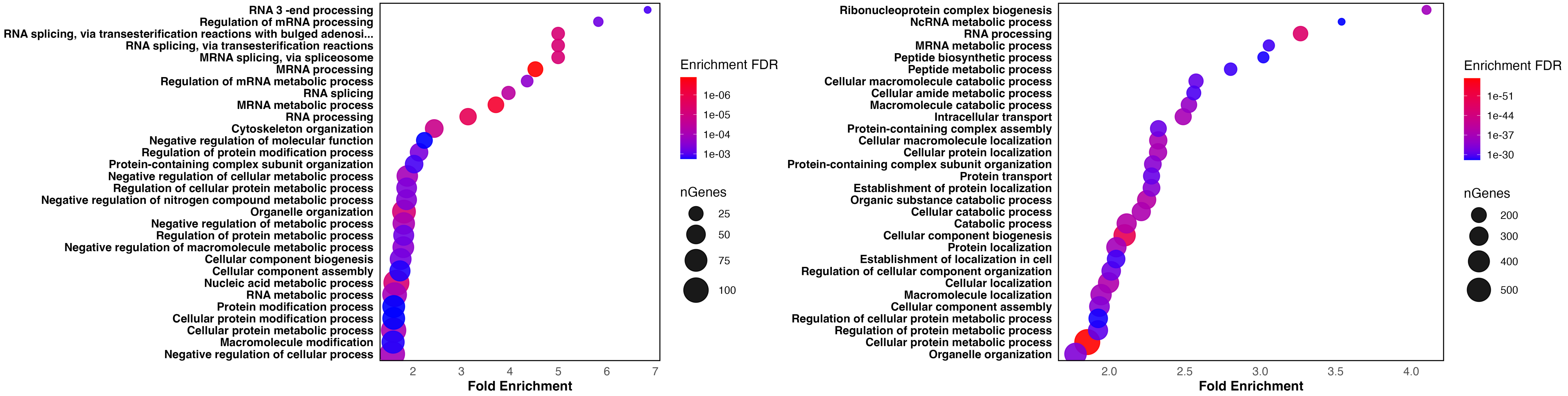

Supplementary Figure S11. Enrichment of biological processes for proteins with altered abundance in the secretome of SV40 MES13 cells exposed to newborn Bothrops jararaca venom for 2 h. Biological enrichment showed for proteins with reduced (A) or increased (B) abundance following exposure to 0.25 μg/mL of venom; reduced (C) or increased (D) abundance at 0.50 μg/mL; reduced (E) or increased (F) abundance at 1.0 μg/mL; reduced (G) or increased (H) abundance at 2.0 μg/mL; and reduced (I) or increased (J) abundance at 4.0 μg/mL of adult *B. jararaca* venom.

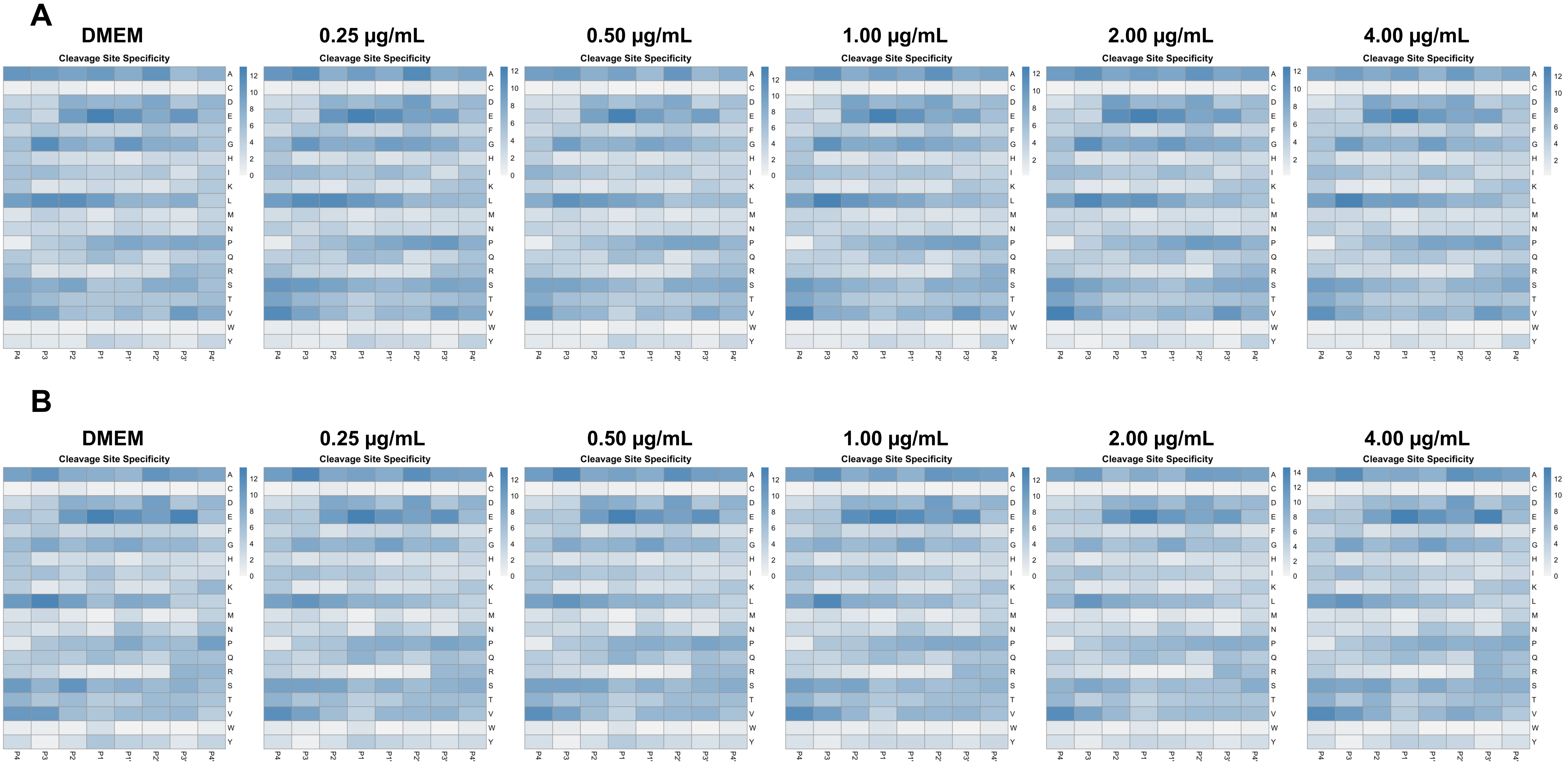

Supplementary Figure S12. Heat maps depicting the relative occurrence of each amino acid residue within proteolytic products spanning positions P4–P4′ in the cellular secretome. The plots show the frequency of amino acid residues in peptides generated from cleaved proteins identified in the secretome of cells incubated with adult (A) or newborn (B) *Bothrops jararaca* venom at different concentrations for 2 h, based on a semi-specific search performed using the same tryptic secretome protein samples.
